# Development and evaluation of triple gene transgenic cotton lines expressing three genes (Cry1Ac-Cry2Ab-EPSPS) for lepidopteran insect pests and herbicide tolerance

**DOI:** 10.1038/s41598-022-22209-w

**Published:** 2022-11-01

**Authors:** Hamid Anees Siddiqui, Shaheen Asad, Rubab Zahra Naqvi, Muhammad Asif, Chengcheng Liu, Xin Liu, Muhammad Farooq, Saifullah Abro, Muhammad Rizwan, Muhammad Arshad, Muhammad Sarwar, Imran Amin, Zahid Mukhtar, Shahid Mansoor

**Affiliations:** 1 Agricultural Biotechnology Division, National Institute for Biotechnology and Genetic Engineering, College Pakistan Institute of Engineering and Applied Sciences (NIBGE-C, PIEAS), Faisalabad, Punjab Pakistan; 2 Department of Biotechnology, University of Sialkot, Sialkot, Pakistan; 3 Beijing Genomics Institute Shenzhen, Shenzhen, China; 4 Plant Breeding and Genetics Division, Nuclear Institute of Agriculture (NIA), Tando Jam, Pakistan

**Keywords:** Molecular biology, Plant sciences

## Abstract

Cotton is an international agricultural commodity and the main cash crop of Pakistan of which quality and quantity are subject to various whims of nature. Climate change, insect pest complex, and weeds are reducing its productivity. Here, we have developed triple gene cotton containing *EPSPS* gene along with two Bt toxin genes *Cry1Ac* and *Cry2Ab* using a strategy where all three genes are cloned in the same T-DNA, followed by successful cotton transformation via Agrobacterium-mediated transformation. This strategy has been developed to help cotton breeders in developing new cultivars by incorporating these genes into the non-transgenic or single Bt (*Cry1Ac*) gene cotton background where all three genes will inherit together. The expression of all three proteins was confirmed through immunostrips and was quantified through enzyme-linked immunosorbent assay (ELISA). The spatio-temporal expression of Bt protein in different parts of triple gene NIBGE cotton plants was determined. Maximum expression was found in leaves followed by seeds and boll rinds. Insect bioassays with cotton bollworms (*Helicoverpa armigera*), armyworms (*Spodoptera litura*), and pink bollworms (*Pectinophora gossypiella*) showed more than 90% mortality. The best performing line (NIBGE-E2) on the basis of spatiotemporal expression, glyphosate assays, and insect mortality data, was used for event characterization by using the genome sequencing approach. The event was successfully characterized and named NIBGE 20-01. A diagnostics test based on event-specific PCR was developed and its ability to distinguish NIBGE 20-01 event from other commercial transgenic cotton events was confirmed. To confirm stable expression of all three proteins in the field conditions, homozygous transgenic lines were grown in the field and the expression was confirmed through immunostrip assays. It was found that all three genes are expressed under field conditions. To show that all three genes are inherited together upon crossing with local elite cotton lines, the F_1_ generation was grown under glasshouse and field conditions. The expression of all three genes was confirmed under field conditions. Our results showed that transgenic cotton with three genes cloned in the same T-DNA can express all genes and can be conveniently transferred into elite cotton lines through a single cross.

## Introduction

Cotton (*Gossypium hirsutum* L.) being an essential fiber-producing crop globally is the main cash crop of Pakistan. It is cultivated on 2.6 million hectares and helps in engaging a substantial number of Pakistani populations towards economic activity. Pakistan is the fifth-largest producer of cotton after China, India, the USA, and Brazil. Cotton has a 0.8% share in the country’s GDP and contributes 4.1% in agricultural value addition^1^. Production of cotton is hampered by many biotic and abiotic factors. Biotic stresses mainly involve the attack of chewing insect pests like cotton bollworms (*Helicoverpa armigera*), spotted bollworms (*Earias insulana*), armyworms (*Spodoptera litura*), and pink bollworms (*Pectinophora gossypiella*)^2^ in addition to sucking insect pests including whiteflies (*Bemisia tabaci*)^3^, aphids and others^4,5^. Many abiotic factors like drought, salinity, high temperature, etc. also contribute to lowering yields. In Pakistan Bt cotton containing the C*ry1Ac* gene was formally approved for cultivation in 2010^6,7^ which showed an increase in the production of cotton. The benefits of Bt cotton introduction among farmers in terms of minimizing pesticide use to control bollworms and decrease in the import bill of pesticides are well documented. Unfortunately, the success was not sustained due to the emergence of several insect pests which resulted in increased use of chemical pesticides. Recently it was found that pink bollworm has developed resistance against Bt cotton expressing the *cry1Ac* gene (Siddiqui et al., data not published) in Pakistan. The resistance breakdown was confirmed by field surveys and field trials. The mutations in the cadherin gene associated with resistance development in pink bollworms have been confirmed. Several known and novel mutations in the cadherin gene were detected in pink bollworms collected from across Pakistan. The extensive use of pesticides to control pink bollworms has not only increased the cost of cotton production but is also causing damage to the environment as well as flared-up whitefly population, probably because of the use of pesticides that promote whiteflies.

Current insect pest management strategies usually involve the use of chemicals such as pesticides which are associated with effects on human health, the environment^8^, and development of pesticide resistance among insect populations^9–11^. The benefits of GM crops are enormous and are one of the top most leading technologies in the world with exceptional adaptability among the farmers. GM crops adaptability among farmers is increasing every year which can be assessed by the fact that in 2005 the area under GM crops was just 90 million hectares^12^, which increased to 191 million hectares in 2018^13^. Bt cotton was first introduced in 1996^14^ and it played a vibrant role in enhancing the production of cotton but with time, production of cotton is threatened as the insects are developing resistance^15–17^ against the Bt cotton^18^ expressing Cry1Ac^19,20^ and Cry2Ab^21–23^. Up till now, a total of fifteen insect species are reported that developed resistance against the Bt toxins^20^ of which only five were reported until 2012^19^. In Pakistan, most of the cultivated Bt cotton varieties had low Bt toxin expression levels and refuge plan was not properly followed which imposed high selection pressure on the chewing insect pests, leading to resistance development^15,24–28^. However, our trials data (data not published) from five majorly cotton growing areas of Punjab and Sindh province showed that double gene Bt cotton expressing Cry1Ac and Cry2Ab is still very much resistant and provides protection against all kinds of chewing insect pests including *H. armigera*, *S. litura,* and *P. gossypiella*.

Weeds are also considered one of the major limiting factors for crop production and contribute significantly to yield losses^29–31^. In addition to the competition with the growing crop in the field, they also act as reservoirs in the field for crop diseases and other insect pests^30^. On average, the losses due to the weeds amount to billions of dollars annually^32^. Manual eradication of weeds is a labor-intensive, time-consuming, and less efficient process^29^. Biotechnology offers exciting opportunities for the modification of crops with an intrinsic herbicide resistance make-up without instigating environmental complications^33–35^.

Event characterization of transgenic plants is the requirement for claiming intellectual property rights (IPRs) and meeting other regulatory requirements for the commercialization of the product^36,37^. It is done by identifying the unknown DNA sequences from the genome flanking the transgene cassette^38^. Different techniques have been used for this purpose in the past including genome walking (GW)^38^ and Southern blotting (SB)^39^. GW and SB are the most widely applied methods for event characterization of GM events, but these are laborious, time-consuming, and expensive. GW in combination with the sequencing approach has also been used for the identification of transgenic events^40,41^. In the past few years, high throughput next-generation sequencing (NGS) technology has emerged as the most promising and efficient approach for the molecular characterization of transgene events. As compared to GW and SB, NGS has been found more sensitive to detecting small insertion/deletions and multiple transgene events in GM crops and other organisms^42–45^.

The objectives of this study were to develop triple gene cotton lines harboring insect and herbicide-resistance genes in one T-DNA and evaluate the spatio-temporal expression of *cry1Ac*, *cry2Ab*, and *EPSPS* genes in different parts of the plants including leaves, bolls, and seeds at different growth stages. Another objective was to check the efficacy of Bt toxins, through insect bioassays with *H. armigera* and *S. litura*, and herbicide tolerance through glyphosate assays using recommended (1900 mL/acre) dose. Another objective was to characterize the transgene integration event of the best-performing line using high throughput NGS technology. Here we have shown that the transgenes are stably expressed under field conditions and can be transferred together in elite local cultivars by a single cross.

## Results

### Construct development

**Figure 1:**
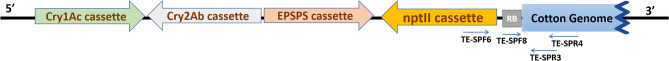
Vector map of triple gene NIBGE cotton. *Cry1Ac* and *Cry2Ab* genes were to provide protection against chewing insect pests mainly bollworms and the *EPSPS* gene was for herbicide tolerance. *Cry1Ac* gene was cloned under 2X 35 promoter; *Cry2Ab* gene was cloned under FMV promoter and *EPSPS* was cloned under CVM promoter.

EPSPS cassette was successfully cloned in pGA482 followed by the cloning of two cassettes of Cry1Ac and Cry2Ab. This final vector pGA482-12ER encompassed triple gene cassettes including Cry1Ac (2 × 35S-Cry1Ac-35S), Cry2Ab (FMV-signal EPSPS-Cry2Ab-G7), and EPSPS (CVM-EPSPS-E9) as shown as vector map (Fig. 1). The sizes of Cry1Ac, Cry2Ab, and EPSPS cassettes were 3.5, 2.8, and 2.6 kb, respectively. The orientation of the cassette in pGA482 was confirmed through restriction analysis (Supplementary Fig. 1). The triple gene construct was named as pGA482-EPSPS-Cry2Ab-Cry1Ac (pGA482-12ER) and was successfully transformed. A confirmed clone of pGA482-12ER was transformed into *Agrobacterium tumefaciens* strain LBA4404 followed by cotton Coker312 transformation. The sequence of the gene construct has been submitted to GenBank under accession number KX880509.

### Development of transgenic Coker312 cotton harboring triple gene construct

Coker312 hypocotyls transformed with pGA482-12ER plasmid successfully started callus formation that led to the development of mature embryos. From mature embryos, T_0_ transgenic plantlets were successfully grown and shifted to the pots in greenhouse conditions. Various T_0_ transgenic lines were developed, and their molecular analysis confirmed the integration of triple gene construct in transgenic plants. After boll setting on T_0_ plants, seeds were harvested from these T_0_ transgenic lines and were further grown to achieve T_1_ generation. These T_1_ generation plants were then grown and NIBGE-E2, NIBGE-E3, and NIBGE-E20 cotton lines were selected for molecular analysis and insect bioassays.

### Transgene analysis of T_1_ triple gene NIBGE cotton plants

Putative transgenic plants of NIBGE triple gene cotton gave amplification of 521, 614, and 510 bp fragments corresponding to *Cry1Ac*, *Cry2Ab,* and *EPSPS* genes, respectively (Supplementary Fig. 2). Expression of the Cry1Ac, Cry2Ab, and EPSPS protein was successfully checked through the immunostrip and ELISA in all the three cotton lines of NIBGE triple gene cotton plants (Supplementary Fig. 3).

### Quantification of Cry2Ab and EPSPS gene expression in T_1_ cotton plants

ELISA of NIBGE cotton lines was performed and gene expression was found comparable to the Monsanto cotton event. For the evaluation of spatial and temporal expression leaves, bolls, and seeds of NIBGE triple gene transgenic cotton lines were used to perform an ELISA assay. Inter and intra-plant variations of Cry2Ab expression was observed in NIBGE triple gene cotton plants. Maximum expression was found in leaves of 30 days old plants with a gradual decrease in the expression with the increasing age of the plant. The results have shown variable expression of Cry2Ab in different parts of the plant. Maximum expression was found in the leaves ranging from 7.3 to 8.77 µg/g of the tissue (Table 1), followed by maturing seeds 4.14–5.56 µg/g of the tissue, and the least expression was observed in boll rinds ranging from 1.7 to 2.7 µg/g of the tissue (Table 2).

**Table 1:** Expression of Cry2Ab in leaves of NIBGE triples gene cotton lines.

DAS	NIBGE triple gene cotton lines			Controls	
	NIBGE-E2	NIBGE-E3	NIBGE-E20	BG II	Coker312
30	8.63 ± 0.8	8.77 ± 0.15	8.32 ± 0.73	8.29 ± 0.43	0 ± 0
60	7.53 ± 0.23	7.81 ± 0.37	7.31 ± 0.79	7.83 ± 0.24	0 ± 0
90	5.96 ± 0.57	6.39 ± 0.23	5.02 ± 0.76	5.74 ± 0.52	0 ± 0
120	4.13 ± 0.43	5.17 ± 0.35	3.21 ± 0.93	4.31 ± 0.31	0 ± 0
135	1.79 ± 0.61	2.31 ± 0.54	1.73 ± 0.71	3.23 ± 0.33	0 ± 0

*DAS* Days after sowing; BGII (MON15985) was used as positive control and Coker312 was used as negative control; ± standard error.

**Table 2:** Cry2Ab expression in boll rind and immature seeds of NIBGE Triple gene cotton.

Sr. no.	Cotton lines	$\frac{{{\text{Cry2Ab}}\;{\text{expression}}\;{\text{in}}\;{\text{boll}}\;{\text{rinds}}}}{{{\text{Cry2Ab}}\;{\text{Conc}}.\;{\mu g}/{\text{g }}\;\left( {{\text{ppm}}} \right) \, \pm {\text{ SE}}}}$	$\frac{{{\text{Cry2Ab}}\;{\text{ expression}}\;{\text{ in}}\;{\text{ immature}}\;{\text{ seeds}}}}{{{\text{Cry2Ab}}\;{\text{Conc}}.\;{\mu g}/{\text{g }}\;\left( {{\text{ppm}}} \right) \, \pm {\text{ SE}}}}$
1	NIBGE-E2	2.7 ± 0.04	5.56 ± 0.96
2	NIBGE-E3	1.7 ± 0.08	4.14 ± 1.14
3	NIBGE-E20	2.2 ± 0.25	5.12 ± 0.87
4	BG II	4.0 ± 0.28	8.72 ± 1.21
5	Coker312	0.00	0.00

*SE* Standard error.

### Insect bioassay of T_1_ triple gene cotton plants

**Figure 2:**
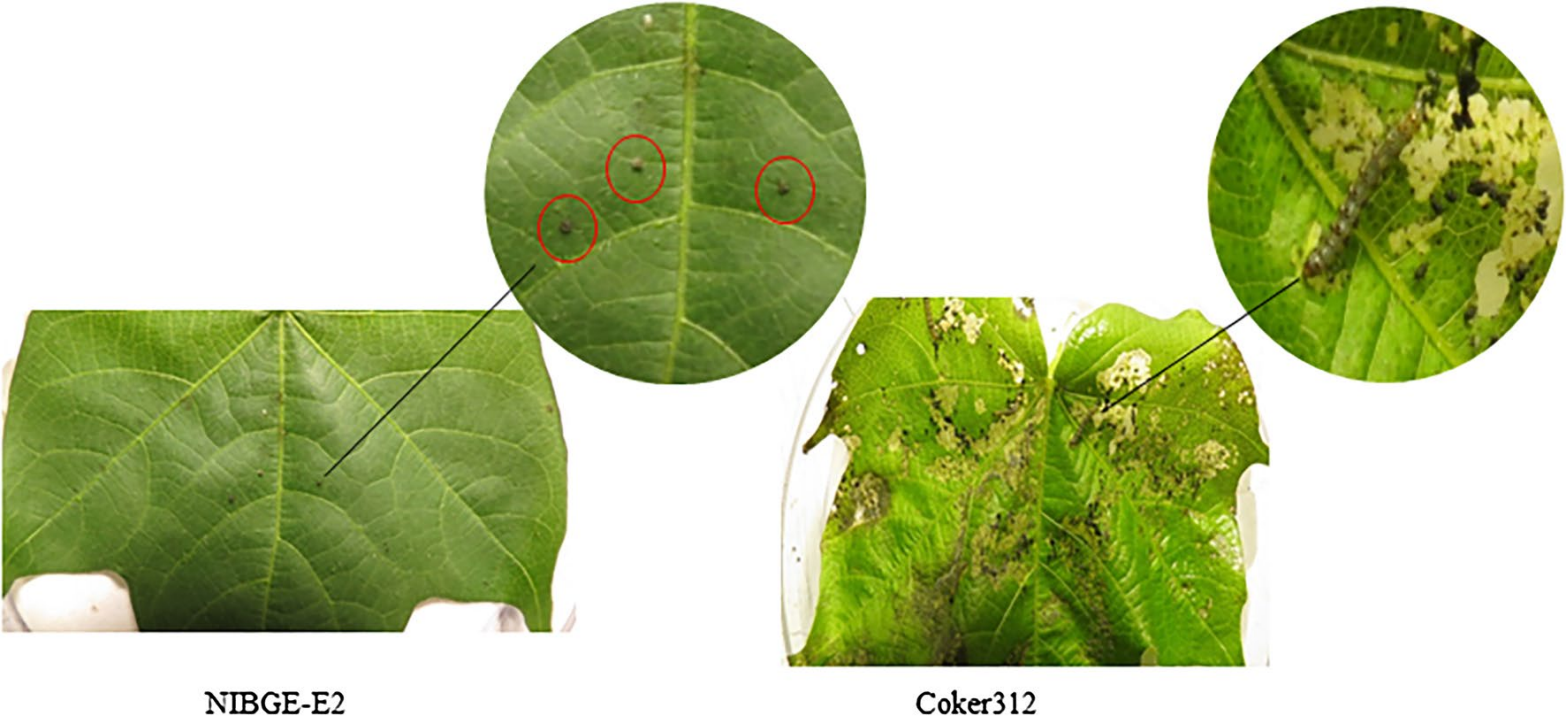
Leaf damage of NIBGE triple gene cotton plants against armyworm. Triple gene NIBGE cotton events (NIBGE-E2, NIBGE E-3 and NIBGE-E20 showed 100% mortality against *S. litura* (armyworm).

Positive transgenic plants were selected through immunostrip and PCR for insect bioassay with armyworm (*Spodoptera litura*). Significant mortality was observed in positive transgenic lines as compared to the non-transgenic Coker312 plants. Insect bioassay of these cotton lines showed 87 to 100% insect mortality using armyworm (*S. litura*). NIBGE-E2, NIBGE-E3, and NIBGE-E20 cotton lines showed 100, 89 and 87% mortality after 96 h, respectively (Fig. 2). Insect mortality data showed variations among the NIBGE triple gene cotton lines as statistically analyzed by ANOVA (p < 0.000). Similarly, the efficacy of triple gene NIBGE cotton against pink bollworm was successfully determined, Data was recorded after 21 days, and bolls were cut to count the number of survivors. NIBGE-E2 and NIBGE-E3 events showed 100% mortality while NIBGE E-20 event showed 86% mortality (Supplementary Figs. 4 and 5). Insect mortality data was in correspondence with gene expression levels of triple gene NIBGE cotton which was determined by ELISA (Fig. 2; Supplementary Figs. 4 and 5). Insect bioassay data were statistically analyzed by ANOVA.

### Glyphosate assay

**Figure 3:**
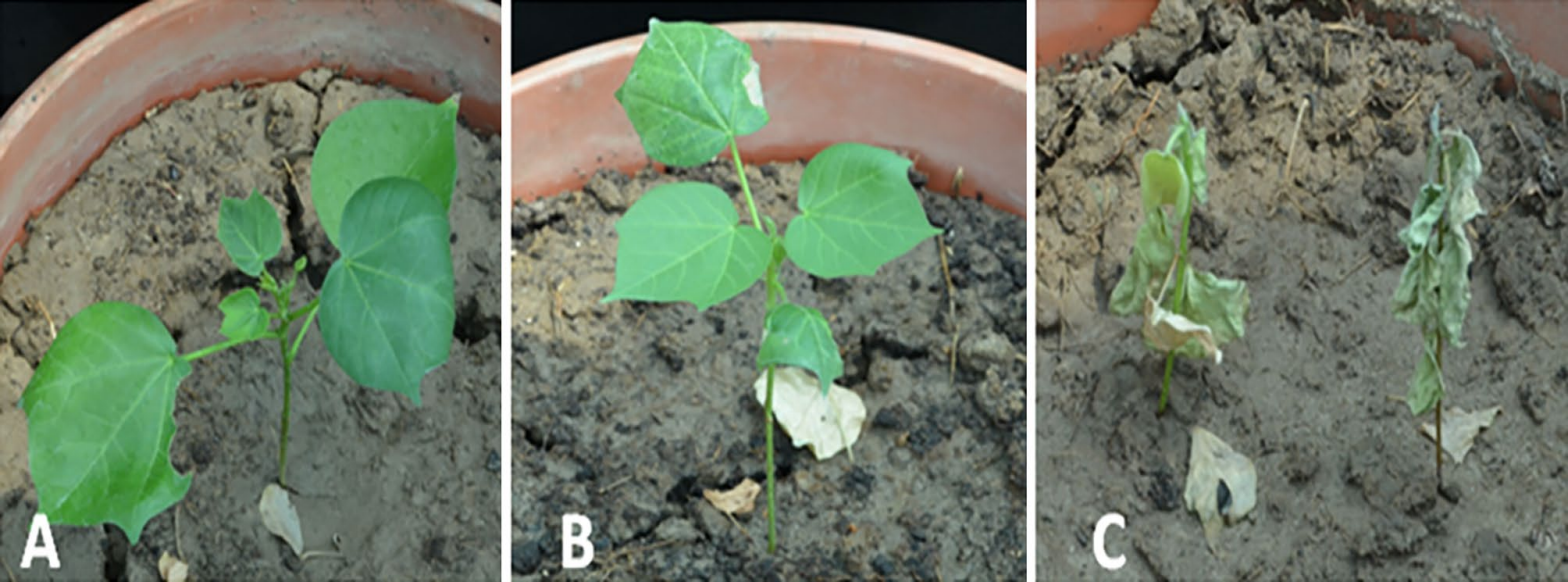
Glyphosate assay of NIBGE triple gene cotton lines at seedling stage (30 DAS) by using herbicide Galaxy @ 1500 ml/acre dose. (**A**) NIBGE Triple Gene Event (E-02), (**B**) RRFBII as a positive control, (**C**) Non-GM Coker-312 as a negative control.

Immunostrip assay showed successful expression of EPSPS in NIBGE triple gene cotton plants, which were then quantified using ELISA. Maximum expression was found in NIBGE-E2 cotton line, followed by NIBGE-E3 and NIBGE-E20. Whole plant glyphosate assays of triple gene cotton showed promising results. NIBGE-E2 showed maximum tolerance against herbicide Galaxy at 1100 mL/acre dose at 35 days old plants. NIBGE-E3 and NIBGE-E20 had shown tolerance against herbicide Galaxy at 900 mL/acre dose. The efficacy of glyphosate assay was consonant with the expression of EPSPS gene in NIBGE triple gene cotton. NIBGE-E3 and NIBGE-E20 cotton lines did not tolerate the 1900 mL/Acre dose but survived on a 900 mL/acre dose. NIBGE-E2 cotton line exhibited significant tolerance to herbicide Galaxy even at 1100 mL/Acre dose (Fig. 3).

### NGS-based event characterization

Whole genome sequencing of NIBGE-E2 cottonresulted in 648.37 M (97.26 Gb) raw bases. After removing low-quality reads, we obtained 638.44 Mb clean reads (95.77 Gb) with a 98.47% clean ratio. The clean reads of the sample had high Q20 (97.73%) and Q30 (92.67%), which showed high sequencing quality. The average GC content was 35.43%. The summary of sequencing results has been shown in (Supplementary Tables 1 and 2). Total clean reads per sample were aligned to the reference genome (Gossypium_hirsutum/HAU-AD1_genome_v1.0_v1.1) using Burrows-Wheeler Aligner (BWA) (Li 2013). The reference genome size is about 2.35 Gb (https://www.cottongen.org/species/Gossypium_hirsutum/HAU-AD1_genome_v1.0_v1.1), contains 2190 scaffold, GC content is 34.3%. The alignment showed that on average, 99.84% mapped successfully and 87.61% mapped uniquely. The duplicate reads were removed from total mapped reads, resulting in about 1.79% duplicate rate and 40.97-fold mean sequencing depth on the whole genome excluding gap regions. We obtained 97.66% of the whole genome excluding gap regions that were covered by at least 1X coverage, 96.68% had at least 4X coverage and 95.34% had at least 10X coverage. In addition, the distributions of per-base sequencing depth and cumulative sequencing depth are shown in (Supplementary Figs. 6–8), respectively. The insert size distribution of paired sequencing reads was plotted (Supplementary Fig. 9). The whole genome sequence of NIBGE triple gene cotton was successfully matched to the NIBGE triple gene construct and flanking regions were identified which was designated as the NIBGE-20-01 cotton event (Supplementary File; Additional Information). Furthermore, the event-specific primers designed for this region detected the specific amplicons as depicted in Table 3 which validated the NIBGE-20-01 cotton event (Supplementary Figs. 10 and 11).

**Table 3:** Primer sequences used for transgene analysis and confirmation of triple gene NIBGE cotton.

Primer name		Primer sequence
NIBGE Cry1Ac specific	CR1BDR5	ATGTCCATAAGGTGAGGTG
	CR1BDF5	TTGCGTGAAGAGATGAGG
NIBGE Cry2Ab Specific	CR2BDR4	ACTTGAGTGGCGTGTATG
	CR2BDF4	CGGTGCTAACTTGTATGC
NIBGE EPSPS specific	EPSR3	GCGAGACGGAGATTTATT
	EPSF3	TGGGTTTGGTTGGTGTTT
Cotton genome specific	TE-SPR1	CCTGTTCCGACTTTGGAAATTCATT
Cotton genome specific	TE-SPR2	TGGATTCCCTGTTCCGACTTTG
Triple gene event specific	TE-SPF1	CGCGCTATATTTTGTTTTCTATCGC
Triple gene event specific	TE-SPF2	ACCGGCAACAGGATTCAATC
Triple gene event specific	TE-SPF3	GTTATGAGGTGACGGTGCTAGG
Triple gene event specific	TE-SPF4	GCCATTTTCCACCATGATATTCGG
Triple gene event specific	TE-SPF5	TCATAGCCGAATAGCCTCTCC

### Field assessment of triple gene NIBGE cotton

Each positive plant for event-specific primers was found to have intact three genes (*Cry1Ac*, *Cry2Ab,* and *EPSPS*) in a single cassette indicating single locus inheritance of the multi-transgenic cassette and proving that multi-transgene cassette can be deployed into elite cotton cultivars instead of single gene transfer. In field conditions, triple gene NIBGE cotton provides significant protection against the pink bollworm and other chewing insect pests including *H. armigera* and *S. litura*. Triple gene NIBGE cotton crossed with FH-1000 (Non-Bt), Bt-30 and Bt-85 showed 100% protection against the chewing insect pests as compared to NIA-Bt-1, NIA-88 (non-Bt. control), and Sohni (Non-Bt check variety) where pink bollworm infestation was observed to be 4.76%, 5.52%, and 7.31%, respectively (Supplementary Table 3).

## Discussion

Genetic engineering provides an opportunity to modify crops to meet the world’s growing challenges of food security and environment-friendly strategies for controlling devastating insect pests. Cotton is one of the most widely cultivated crops in the world with a huge impact on the economy, especially in developing countries. Bt cotton was initially introduced in 1996 containing the *Cry1Ac* gene, providing resistance against the chewing insect pests and revolutionized pest control entailing a significant increase in the yields. However, in recent years pink bollworm has developed resistance against Cry1Ac protein. We identified mutations in the cadherin gene of pink bollworm that are likely to be involved in the resistance development (data not shown). Moreover, we showed that transgenic cotton plants expressing both Cry1Ac and Cry2Ab are resistant to pink bollworm under field and glasshouse conditions (data not shown).

In this study, we have designed a triple gene construct containing *Cry1Ac* and *Cry2Ab* genes for providing resistance against the chewing insect pests and EPSPS gene for herbicide tolerance. All three genes are cloned in the same T-DNA^5^ under different promoters and were transformed into cotton. This strategy was developed to help breeders in developing new cotton cultivars by incorporating these genes into the non-transgenic cotton cultivars or cotton cultivars containing a single Bt gene *Cry1Ac* (Mon531), where all three genes are inherited together. Previously, transgenic cotton with herbicide and insect resistance cotton varieties have been developed but those were developed through traditional breeding techniques between the herbicide tolerant and insect resistant cotton varieties.

To control the chewing insect pests, especially bollworms it is highly indispensable to have a sustainable expression of Bt toxins Cry1Ac and Cry2Ab in the vulnerable parts of the plant. It has been shown that the level of toxin declines with the age of the plant and even drops below the critical level at later stages which imposes high selection pressure on the insect pests to develop resistance against toxins^24,46,47^. In this study, we have quantified the spatio-temporal expression levels of the toxin in leaves, maturing seeds, and boll rinds of the green bolls which are considered as the most vulnerable parts of the plant, especially bollworms. The highest level of the toxin was found in leaves at the seedling stage followed by the maturing seeds and the lowest level of the toxin was found in the boll rinds of green bolls which shows variations throughout the growing season of the plant and was found variable among the triple gene cotton events (NIBGE-E2, NIBGE-E3, and NIBGE-E20).

Apart from the insect pests, weeds are another area that have gained significance due to the higher cost of labor for manual weeding and climate change that is resulting in unpredictable rain patterns in cotton growing areas. Therefore, cause significant loss in the crop yield resulting in decline yield. In the past weeds culminated through mechanical, cultural, and biological means which is a difficult and time-consuming process^32,33,35^. The discovery of glyphosate and its use in mid-nineties contributed vastly to the increase of world food production. In this study, a triple gene cotton containing *EPSPS* gene along with pest resistance Bt toxin genes *Cry1Ac* and *Cry2Ab* have been developed. The expression of the EPSPS gene in triple gene NIBGE cotton events was determined. The highest expression of the *EPSPS* gene was found in the NIBGE-E2 event while the lowest in the NIBGE-E20 event. Efficacy of *EPSPS* gene was confirmed through glyphosate assay at seedling stage and NIBGE triple gene cotton event E2 have shown maximum tolerance against the herbicide Galaxy @1100 mL/acre dose. E3 and E20 NIBGE triple gene cotton events were mildly tolerant to herbicide Galaxy @1100 mL/acre dose and were fully tolerant @900 mL/acre dose.

Our objective was to develop triple gene cotton containing insect and herbicide resistance genes under different promoters for a sustained expression of genes and in a single T-DNA which will be helpful for breeders for introgression in elite cultivars. Bollworms, *H. armigera,* and *S. litura* mainly feed on seeds more preferably than foliage and consistent expression of Cry1Ac is necessary for the developing seeds^6^. We selected the best performing event and used the whole genome sequencing approach for event characterization^41–45^ and the development of PCR based event-specific diagnostic tool. We showed that the diagnostics can distinguish NIBGE events from previously reported events. These diagnostics will not only help in securing intellectual property rights but also help breeders in the selection of desirable plants in the selection process. In the future tissue-specific promoter like alpha globulin and nodulin-like promoters may also be used for a sustainable expression of Bt toxins in seeds and reproductive parts, respectively. Apart from transgenic technology where two Bt genes are expressed together, other practices like mating disruptions or sterile insects must be used for effective and sustainable control of pink bollworms and other bollworms^48–51^.

## Material and methods

### Triple gene (*Cry1Ac* + *Cry2Ab* + *EPSPS*) construct development

All three genes (*Cry1Ac*, *Cry2Ab,* and *EPSPS*) were codon optimized for the cotton genome and were commercially synthesized. EPSPS full cassette (about 2.6 kb) was restricted with *Xba*I (Thermo Fisher Scientific ER0682) and *Hind*III (Thermo Fisher Scientific ER0502) from EPSPS cassette cloned in pBlue vector^5^. Binary vector pGA482 was also restricted using the pair of *Xba*I (Thermo Fisher Scientific ER0682) and *Hind*III (Thermo Fisher Scientific ER0502) and was ligated to EPSPS full cassette forming the clone named EPSPS-pGA482. EPSPS-pGA482 was restricted with *Hpa*I (Thermo Fisher Scientific ER1031; blunt end cutter) and ligated to *Swa*I (Thermo Fischer Scientific ER1241) restricted double gene (*Cry1Ac* + *Cry2Ab*) construct that was already developed as described by Siddiqui et al.^6^. After screening, a positive clone was named pGA482-12ER, and orientations of genes were verified by restriction digestion.

### Cotton transformation

Triple gene vector, pGA482-12ER having *Cry1Ac-Cry2Ab-EPSPS* cassettes was transformed into *Agrobacterium* strain LBA4404, and clones were confirmed through colony PCR. The confirmed clone was further used for the transformation of cotton *var.* Coker312. Hypocotyls were excised from 7 days old plantlets of Coker312 and were immersed into *Agrobacterium* culture of the pGA482-12ER plasmid. These hypocotyl cuttings were dipped in bacterial culture and were occasionally shaken. Afterward explants were dried on a blotting paper, followed by their transfer onto co-cultivation MS medium (MSP09-100LT, Caisson Labs-USA), where for two days; these were incubated in dark at 26 ± 2 °C. Then for callus initiation, these hypocotyls were further shifted to the callus induction medium. After callus formation, the calli were placed on an embryo maturation medium. Once embryos started maturing, these were shifted to germination medium followed by shifting onto shooting and rooting media in jars. Throughout the process, the shooting and rooting medium was supplemented with 50 mg/L kanamycin (Carl ROTH) and 200 mg/L cefotaxime. The plantlets with fully established roots were shifted to pots containing peat moss and then after hardening was shifted to pots having soil.

### Molecular analysis of transgenic cotton plants

Molecular analysis of putative transgenic cotton plants was made through immunostrip test and polymerase chain reaction (PCR). For the immunostrip test (Catalog No. AS-046-LS, Envirologix USA), 20–25 mg of leaf tissue was brought to lab in a 1.5 mL microcentrifuge tube and placed on ice. Leaf was ground in 300 µL of extraction buffer given in the kit (Catalog No. AS-046-LS, Envirologix USA) using mortar and pestle and then centrifuged at 13,000 rpm for one minute. The supernatant containing protein was collected in a separate sterile 1.5 mL microcentrifuge tube, and an immunostrip for the triple gene (*Cry1Ac, Cry2Ab,* and *EPSPS*) was inserted in the supernatant and allowed to stay at room temperature to develop the required bands. Positive plants were selected and further evaluated through standard PCR using gene-specific primers (Table 4). DNA was extracted from the positive transgenic plants through CTAB (Cetyl Trimethyl Ammonium Bromide) method^52^. For PCR amplification, NIBGE gene-specific primers for *Cry1Ac*, *Cry2Ab*, and *EPSPS* were used. Monsanto event-specific primers for Bollgard II and Mon531 were used to rule out the presence of Monsanto events in NIBGE triple gene cotton lines. SadI primers^6^ were used as an internal control to check the quality of DNA. PCR reaction mixture was prepared using 7.5 µL Dream Taq Green Master Mix (Cat No. K1081, Thermo Fisher Scientific), 5 µL (50 ng/µL) genomic DNA from cotton, 1 µL (10 µM) of each gene, and event-specific primer^53^ and 11.5 µL of deionized water was used to make total reaction volume of 25 µL. A Bio-Rad PCR thermal cycler (C1000 Touch™) was used with the following PCR profile in which initial denaturation was done at 95 °C for 5 min, then 40 cycles with denaturation at 95 °C for 40 s, annealing at 55 °C for 40 s and then extension at 72 °C for 1 min, and with a final extension of 10 min at 72 °C. Primer sequences for nptII, Sad1, MON15985, MON531, and MON1445 were retrieved from the GMO Detection Method Database (GMDD; www.gmdd.shgmo.org) and the published literature^53^.

**Table 4:** Immunostrip and PCR-based transgene analysis of triple gene NIBGE cotton lines.

Samples	Immuno-strip test			PCR analysis using specific primers					
	Cry1Ac	Cry2Ab	EPSPS	SadI	EPSPS	NIBGE Cry1Ac	NIBGE Cry2Ab	BG	BGII
NIBGE-E02	+	+	+	+	+	+	+	−	−
NIBGE-E03	+	+	+	+	+	+	+	−	−
NIBGE-E20	+	+	+	+	+	+	+	−	−
Coker312	−	−	−	+	−	−	−	−	−
BGII	+	+	−	+	−	−	−	+	+

BGII, MON15985; NT, not tested; (+), presence of gene and (−), absence of gene.

### Quantification of Bt gene Cry2Ab

Spatio-temporal expression of Cry2Ab was quantified through ELISA (enzyme-linked immunosorbent assay) using an ELISA kit (Catalog No. AP 005, Envirologix USA). Ten seeds from each of three triple gene cotton lines (NIBGE-E2, NIBGE-E3, and NIBGE-E20) were grown in the greenhouse under controlled conditions (temperature, humidity) along with controls including Coker312 as negative control and Monsanto event Bollgard II (Mon15985) as a comparative control. After the identification of positive transgenic cotton plants through immunostrip and PCR analysis, ELISA was performed on different parts of the plant like the leaf, boll rind, and seeds. 10–15 mg of tissue was taken in a 1.5 mL microcentrifuge tube submerged in ice was taken and brought to the lab. ELISA was performed according to the given protocol with the kit (Cat No. AP 005, Envirologix, USA) and Cry2Ab expression was quantified and shown in µg/g of tissue weight by drawing a standard curve that was drawn using OD values from calibrators.

### Glyphosate assay

Glyphosate assay of NIBGE triple gene cotton lines was performed at the seedling stage by using different doses of herbicide Galaxy i.e., @1900, 1500, and 1100 mL/acre dose. The herbicide solution was prepared and sprayed on thirty days old plants carefully. Coker312 and wild grass were used as negative controls and the equal dose was also sprayed on Coker312 and wild grass. The experiment was conducted under controlled conditions in a greenhouse and the data was recorded after 14 days of spraying.

### Insect bioassays of transgenic cotton

To evaluate the efficacy of transgenic cotton lines insect bioassay was conducted with armyworm (*S. litura*). Three transgenic cotton lines (NIBGE-E2, NIBGE-E3, and NIBGE-E20) were used to check the efficacy of Bt toxins (Cry1Ac and Cry2Ab). Coker312 was used as a negative control and Bollgard II cotton (Mon15985) was used as a positive control in this experiment. Five plants of each line were selected randomly and three leaves from each plant were taken to be used in the bioassay. Each leaf was placed on a plate on filter paper and the leaf petiole was wrapped with wet cotton to keep the leaf fresh. Each leaf was exposed to five first instar larvae. During the experiment temperature and relative humidity were maintained between 25 ± 1 and 50–70%, respectively. Insect bioassay was performed twice with the same lines and controls entailing non-transgenic Coker312 as a negative control and Bollgard II cotton (Mon15985) as a positive control. Data was recorded to count the mortality rate of larvae every 24 h for five consecutive days until the completion of the bioassay. For calculating the difference in mortality between the transgenic and non-transgenic cotton lines, data was properly analyzed statistically using the analysis of variance (ANOVA) and least significant difference test (LSD).

### Boll bioassay against pink bollworm

Boll bioassay of triple gene NIBGE cotton was conducted at NIBGE. Seeds of triple gene NIBGE cotton events (NIBGE-E02, NIBGE-E03, and NIBGE-E20) were grown in a greenhouse under controlled conditions. Once germinated, transgenic plants were verified with immunostrip (Catalog No. AS-046-LS, Envirologix, USA) and PCR using gene-specific primers for the Bt genes (*Cry1Ac* and *Cry2Ab*). Plants were grown and maintained in the greenhouse of NIBGE, without applying any insecticide. Ten days old bolls were collected from the plants and brought to the insect molecular biology lab of NIBGE. Bolls were briefly washed with running water and blot dry. Five bolls from each event were placed in a container. The container was closed with cloth lining to increase the ventilation and to control the fungal growth due to moisture as much as possible. Fifteen first instar larvae were carefully placed in each container using a fine brush. Bolls from the non-Bt cotton (Coker-312) and BGII (Mon15985) were used as a negative and positive control, respectively. After 22 days, all bolls from Bt (triple gene NIBGE cotton & BGII) and non-Bt (Coker312) were cut, and a number of survivors were counted inside the boll. The survival rate was calculated as the number of survivors divided by the total number of larvae placed in the container.

### NGS-based event characterization of triple gene NIBGE cotton

Based on the gene expression and bioassays, the best performing transgenic cotton line (NIBGE-E2) was selected and further used for event characterization using NGS technology. Highly purified good quality genomic DNA was isolated from confirmed -triple gene NIBGE-E2 cotton plant by using PureLink™ genomic plant DNA purification kit (Catalog No. K183001, Thermo Fisher Scientific) and single replicate of NIBGE-E2 cotton was sequenced on BGISEQ-500 platform. Sequencing data in the form of paired-end reads stored in FASTQ format was obtained and data filtering was done to remove adapters, noisy or low-quality base ratio reads, and reads with unknown 'N' bases. The cleaned data were aligned to the reference cotton genome using Burrows-Wheeler Aligner (BWA) (Li 2013); followed by alignment with the triple gene NIBGE cotton construct sequence. The alignment was performed in CLC Genomics Workbench to detect the event in the genome of NIBGE triple gene cotton (. For NGS-based detected events in triple gene cotton, the event-specific primers (Supplementary Table 2) were designed from the junction region of the cotton genome and triple gene construct to validate the results. Event-specific primers were thoroughly evaluated and confirmed to be different from Monsanto cotton BGI (Mon531) and BGII (Mon15985) events.

### Field evaluation of NIBGE triple gene transgenic cotton lines

The research work was conducted at Plant Breeding and Genetics Division, Nuclear Institute of Agriculture (NIA), Tando Jam, Pakistan during the Kharif seasons of 2019–2020 and 2020–2021. The experimental material consisted of triple gene (NIBGE-20-01) cotton developed at NIBGE Faisalabad, Non-Bt and Bt controls, and different crosses which were developed at NIA, Tando Jam.

For crossing, unopened flowers commonly known as candles or buds were hand emasculated in the evening and covered with butter paper bags. Male parent flowers were also covered with butter paper bags to avoid insect-intervened pollen contamination. Pollination of flowers was carried out in the morning (10:00 AM) and pollinated flowers were again covered with butter paper bags. At maturity, seed cotton was picked from all cross combinations. Ginning was performed and F_0_ seeds were collected for evaluation in the F_1_ generation. During Kharif 2020-21, F_1_ genetic material along with controls was planted in field conditions. Each plant in the F_1_ generation was tested for the presence of a triple gene cassette using immunostrip assay (Catalog No. AS-046-LS, Envirologix, USA) and through PCR by using NIBGE triple gene cotton event specific primers. Data on single plants were recorded for pink bollworm infestation (%) and seed cotton yield (g/plant). The percent infestation by pink bollworm was estimated by collecting boll samples from plants. Green susceptible bolls (bolls that can be easily pressed between the index finger and thumb) were collected from each plant, dissected, and examined for the presence and damage caused by pink bollworm larvae. Two observations were recorded with 15 days intervals in peak infestation period i.e., during September.

Percentage infestation of pink bollworm was calculated by using the formula:

$$\mathrm{Infestation }\left(\mathrm{\%}\right)=\frac{\mathrm{No.} \,\mathrm{ of\, damaged\, fruiting\, parts}}{\mathrm{Total\, no}.\mathrm{ \,of \,fruiting \,parts}}\times 100$$

### Ethical statement

The study was approved by the Institutional Biosafety Committee (IBC) of the National Institute for Biotechnology and Genetic Engineering. The approval was given under the number IBC No. NIBG-49-112. All genetic manipulation in plants is approved by Institutional Biosafety Committee, duly registered with National Biosafety Committee (NBC). The case NIBG-49-112 was recommended by Technical Advisory Committee and approved by NBC.

### Handling of plants/seeds

Handling of plants/seeds were carried out by relevant guidelines and regulations.

## Supplementary Information

Supplementary Information.

## Data Availability

The SRA datasets generated and/or analyzed during the current study have been submitted to NCBI with the accession number PRJNA801917 and can be accessed through the following link https://www.ncbi.nlm.nih.gov/sra/PRJNA801917.
