# Supplementary material for: Development and evaluation of triple gene transgenic cotton lines expressing three genes (Cry1Ac-Cry2Ab-EPSPS) for lepidopteran insect pests and herbicide tolerance
Source: Sci Rep. 2022 Nov 1;12:18422. doi: 10.1038/s41598-022-22209-w (PMC9626562; doi:10.1038/s41598-022-22209-w)
Supplement: Supplementary file 1 — Supplementary Information. [file 41598_2022_22209_MOESM1_ESM.docx]

**Development and evaluation of triple gene transgenic cotton lines expressing three genes (Cry1Ac-Cry2Ab-EPSPS) for lepidopteran insect pests and herbicide tolerance.**

**Hamid Anees Siddiqui^1, †^, Shaheen Asad^1, †^, Rubab Zahra Naqvi^1 †^, Muhammad**

**Asif^1^, Chengcheng Liu^2^, Xin Liu^2^, Muhammad Farooq^1^, Saifullah Abro^3^,**

**Muhammad Rizwan^3^, Muhammad Arshad^1^, Muhammad Sarwar^1^, Imran Amin^1^,**  **Zahid Mukhtar^1*^, and Shahid Mansoor^1*^**

**^1^**Agricultural Biotechnology Division, National Institute for Biotechnology and Genetic Engineering, College Pakistan Institute of Engineering and Applied Sciences (NIBGE-C, PIEAS), Faisalabad, Punjab, Pakistan

**^2^**Beijing Genomics Institute Shenzhen, China

**^3^**Plant Breeding and Genetics Division, Nuclear Institute of Agriculture (NIA), Tando Jam, Pakistan

**^†^**These authors contributed equally to this work

***Shahid Mansoor** shahidmansoor7@gmail.com

***Zahid Mukhtar** zahidmukhtar@yahoo.com **Supplementary Tables.**

**Supplementary Table 1 |** Summary of whole genome sequencing data

| Sample | Raw  reads  (M) | Raw  bases  (Gb) | Clean reads  (M) | | Clean  Bases  (Gb) | | Clean ratio  (%) | | Q20  (%) | | Q30    (%) | GC  content  (%) | |
| --- | --- | --- | --- | --- | --- | --- | --- | --- | --- | --- | --- | --- | --- |
| SIDE2 | 648.37 | 97.26 | 638.44 | | 95.77 | | 98.47 | | 97.73 | | 92.67 | 35.43 | |
| **Supplementary Table 2 \|** Summar  y statistics | | | | | of alignment | |  | |  | |  |  | |
| Mappin Uniqu Duplicat  Sampl g rate e rate e rate h rate g depth e  (%) (%) (%) | | | | Mismatc  (%) | | Sequencin  (X) | | Coverag e (%) | | Coverag e (10X) | | | Coverag e (20X) |
| SIDE  99.84 87.61 1.79  2 | | | | 0.48 | | 40.97 | | 97.66 | | 95.34 | | | 92.28 |

**Supplementary Table 3 |** Field evaluation of F_1_ generation (single plants) for the presence of triple gene, bollworm infestation and seed cotton yield

| Cross combinations and Controls | Inheritance of triple gene | | | Pink bollworm infestation (%) |
| --- | --- | --- | --- | --- |
|  | Cry1Ac | Cry2Ab | EPSPS |  |
| FH-1000 (non-Bt) × NIBGE-20-01 | **+** | **+** | **+** | 0.00 |
| Bt-30 × NIBGE-20-01 | **+** | **+** | **+** | 0.00 |
| Bt-85 × NIBGE-20-01 | **+** | **+** | **+** | 0.00 |
| NIA-Bt-1 (Bt. control) | **+** | **-** | **-** | 4.76 |
| NIA-88 (non-Bt. control) | **-** | **-** | **-** | 5.52 |
| Sohni (non-Bt. check variety) | **-** | **-** | **-** | 7.31 |

**Supplementary Figures**


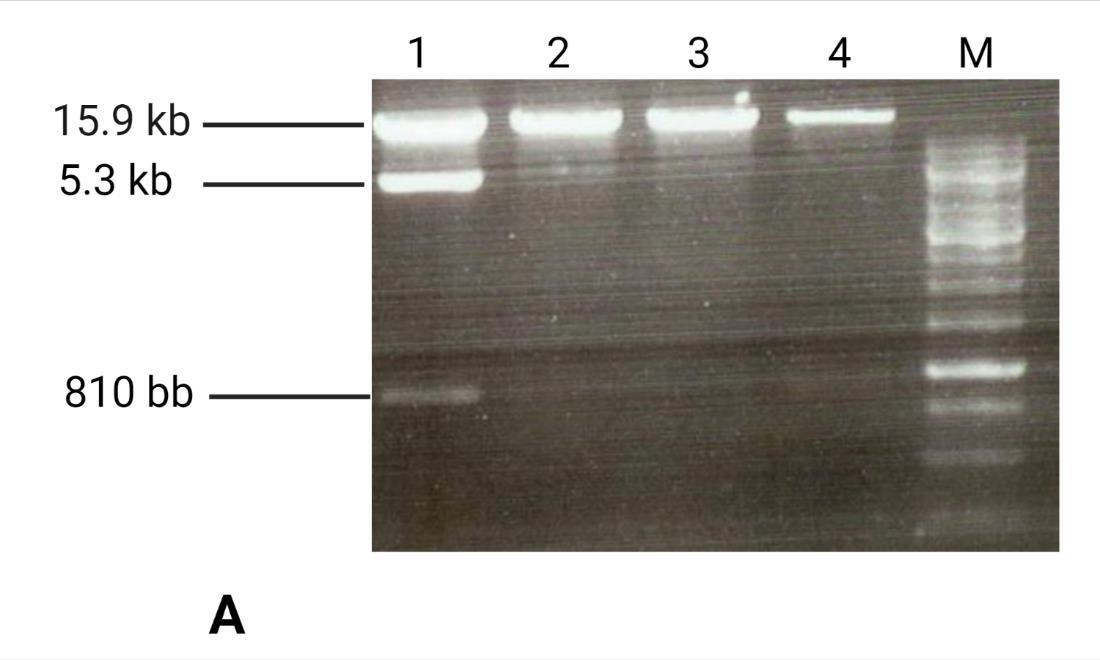


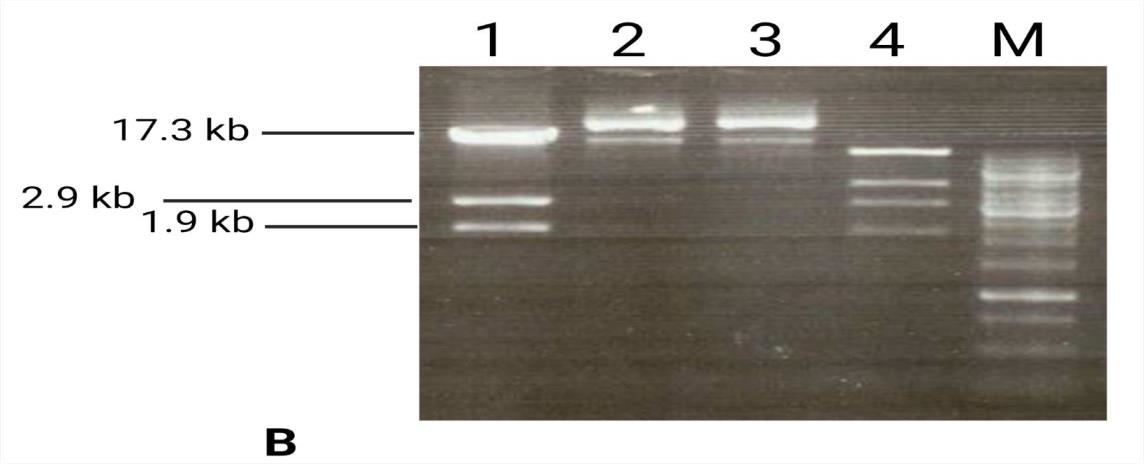

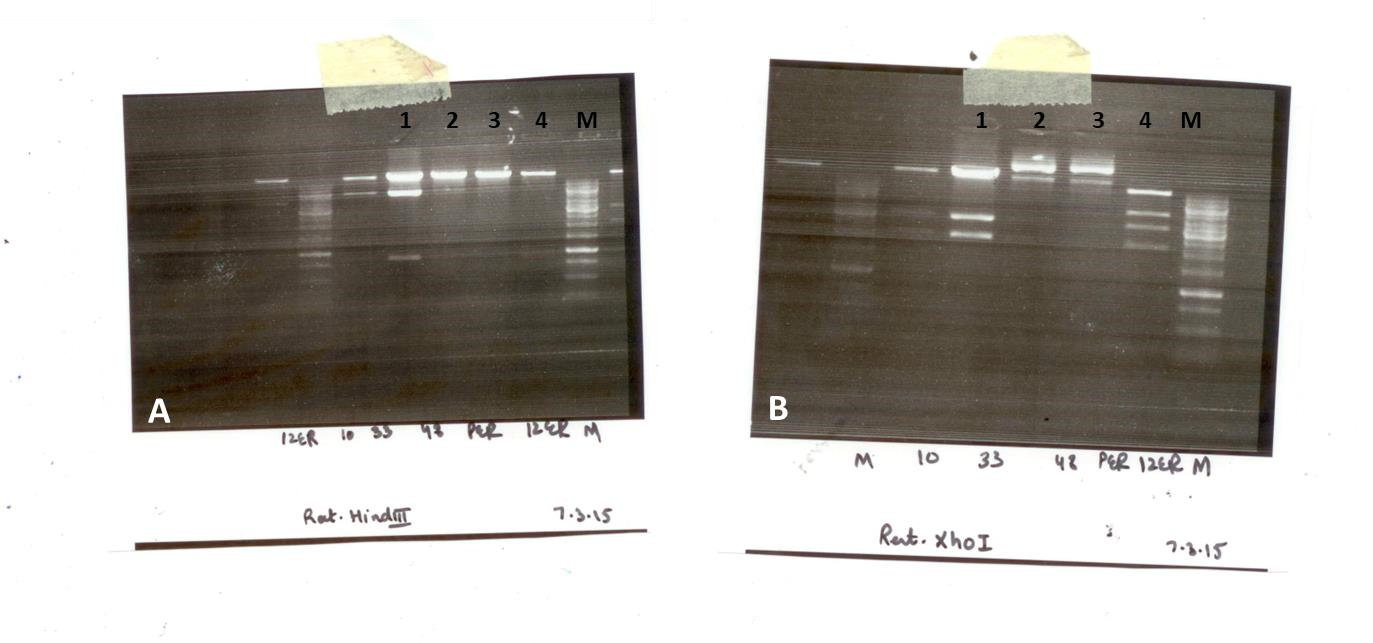


**C**


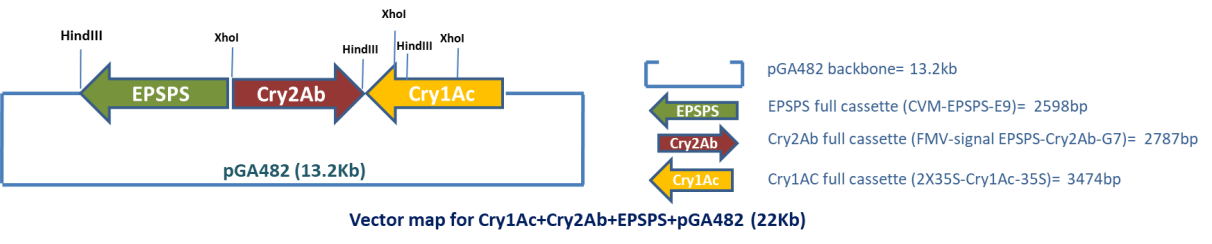


**D**

**Supplementary Figure 1 | A** Orientation confirmation of Cry1Ac+Cry2Ab+EPSPS (12ER) in pGA482 with HindIII restriction;1**:** pGA482+Cry1Ac+Cry2Ab+EPSPS giving desired fragments of 810bp, 5.3kb and 15.9kb; **2:** pGA482+full EPSPS cassette is linearized as has only single HindIII site **3:** pGA482+full EPSPS cassette is linearized as has only single HindIII site **4:** Cry1Ac+Cry2Ab+EPSPS +psb187 giving only a band of 810bp (not very visible in gel due to low intensity); **M:** Marker 1 Kb **B:** Confirmation of Cry1Ac+Cry2Ab+EPSPS (12ER) in pGA482 with XhoI restriction**: 1:** pGA482+Cry1Ac+Cry2Ab+EPSPS giving desired fragments of 1.9kb, 2.9kb, and 17.3kb; **2:** pGA482+full EPSPS cassette is uncut as no XhoI site in the vector ; **3:** pGA482+full EPSPS cassette is uncut as no XhoI site in the vector **4:** Cry1Ac+Cry2Ab+EPSPS +psb187 giving 1.9kb, 2.9kb, 4.5kb and 8.8kb **M:** Marker 1 Kb , **C:** Original images of gels shown in A & B, and **D** represents the vector map of pGA482+Cry1Ac+Cry2Ab+EPSPS showing respective HindIII and XhoI sites used for orientation confirmation.


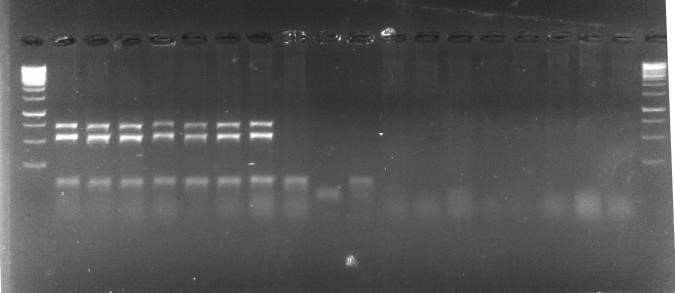

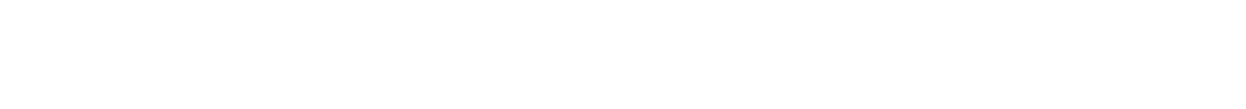

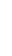

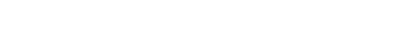


M 1 2 3 4 5


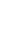

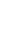


6


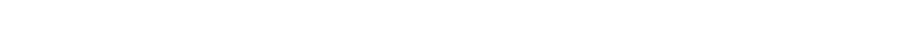


8 9 10 11 12 13 14 15 16 17 18 M

7


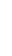

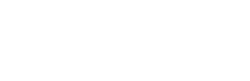

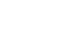


SadI


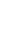

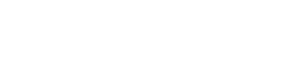

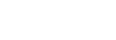


Cry1Ac


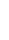

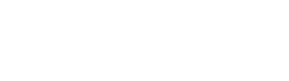

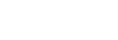


Cry2Ab


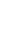

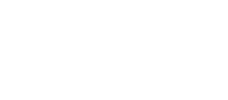

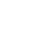


**A**


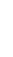

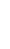

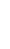

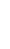

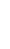

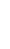

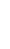

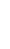

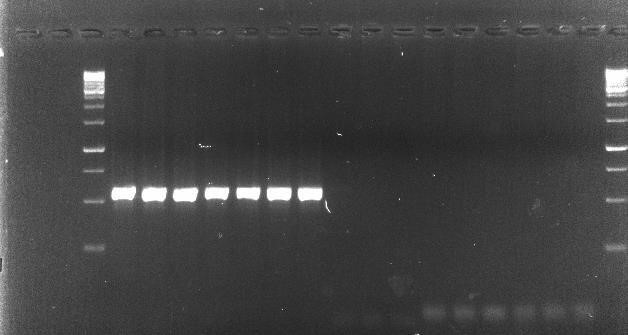

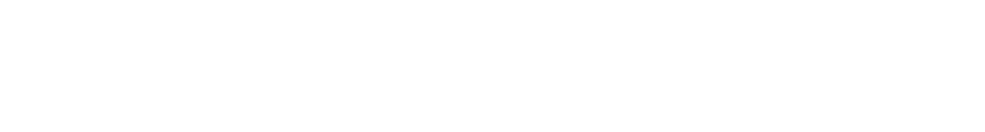

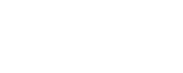


M 1 2


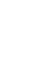


3


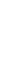

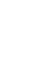


4


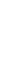

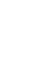


5


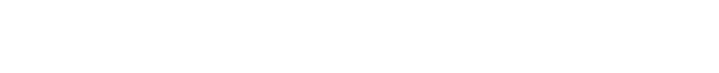


6

7 8 9 10 11 12 13 14M


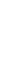

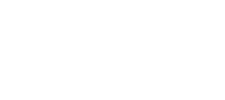

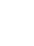


**B**


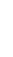


**Supplementary Figure 2 |** Molecular analysis of NIBGE triple gene cotton lines using PCR. **A.** 1-7, lower shows the amplification of sadI (106 bp), middle row shows the amplification of Cry1Ac (521 bp) and upper shows the amplification of Cry2Ab (614 bp) by using NIBGE gene specific primers; 9,10 for non-GM Coker 312 as negative control; 11, 12, 13 for Mon531 using Mon531 event specific primers; 16, 17, 18 for BG II (Mon15985) using BGII event specific primers. **B.** 1-7 for NIBGE EPSPS gene (568 bp) using NIBGE EPSPS gene specific primer; 8,9,10 for non-GM Coker 312 as negative control; 11-14 for RRF using Monsanto event specific primers.

**E02**  **E03**  **E20**  **Coker**


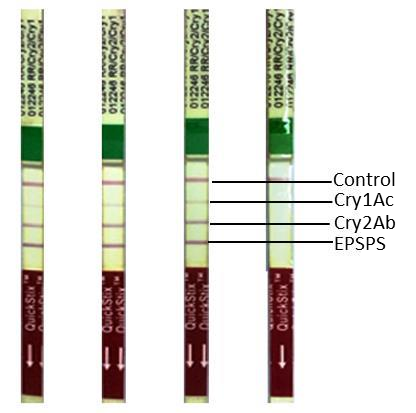

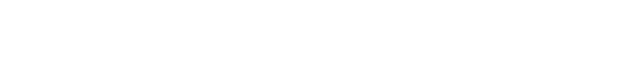

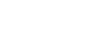

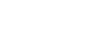

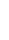

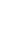

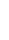

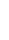

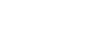

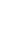

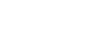

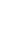

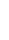

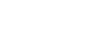

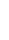


**Supplementary Figure 3 |** Immunostrip assay of NIBGE triple gene cotton lines (Triple gene). E2, E3, and E20 are NIBGE triple gene cotton events showing expression of *Cry1Ac*, *Cry2Ab* and *EPSPS* genes and non-GM Coker 312 was used as a negative control.


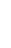

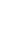


0


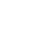


20


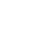


40


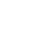


60


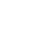


80


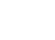


100


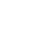


E2


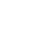


E3


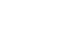


E20


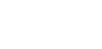


BG II


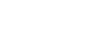


Coker


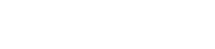

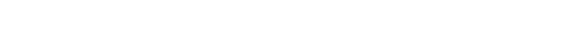


NIBGE Triple gene cotton lines


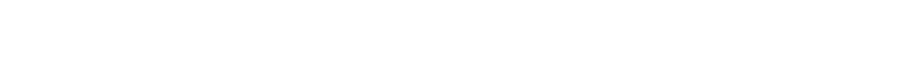


Boll bioassay of NIBGE triple gene cotton


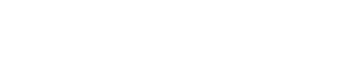


lines using PBW

**Supplementary Figure 4 |** Mortality rate of pink bollworm on bolls from triple gene NIBGE cotton plants. Boll bioassay of triple gene NIBGE cotton was conducted to show the efficacy of Bt genes. In this experiment NIBGE-E2, NIBGE-E3 and NIBGE-E20 were used as triple gene NIBGE cotton while Coker312 and BGII were used as negative and positive control, respectively. NIBGE-E2 and NIBGE-E3 show 100% mortality and 86% mortality was observed in NIBGE-E3 triple gene NIBGE cotton events. Error bars represent the standard error among the replicates.

**Supplementary Figure 5 |** Boll damage from Boll bioassay of triple gene NIBGE cotton plants using pink bollworm. Triple gene NIBGE cotton event E2 showed 100% mortality. BGII was used as positive control which also showed 100% mortality while Coker312 was used as negative control and it showed only 13% mortality.


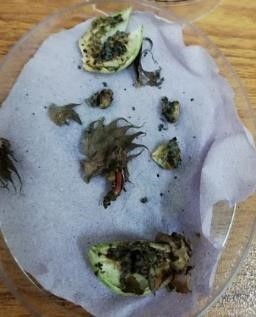

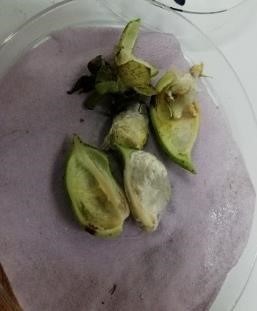

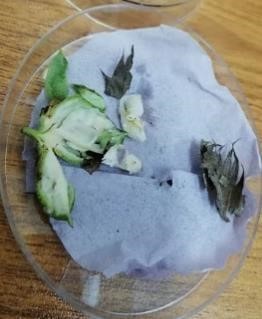

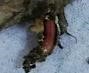

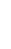

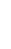


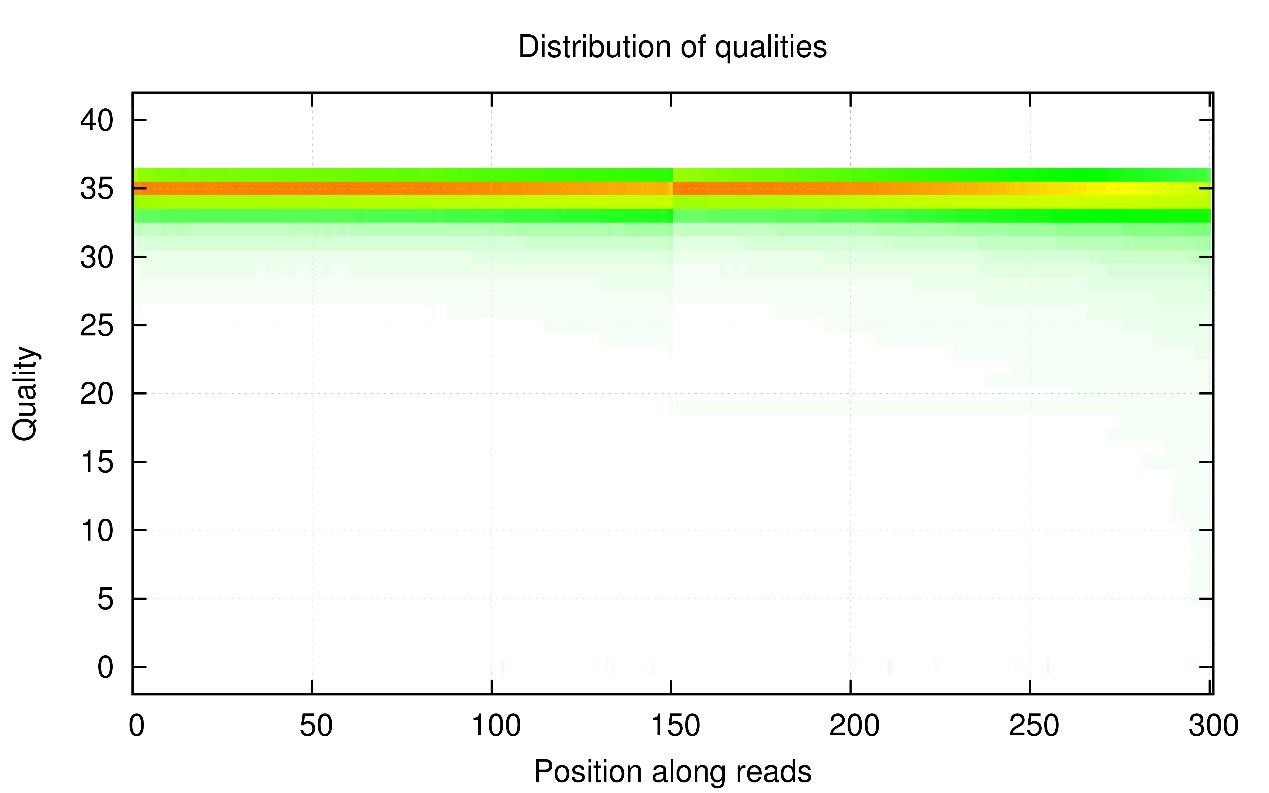


**Supplementary Figure 6 |** Distribution of base quality scores on clean reads. X-axis is positions along reads. Y-axis is quality value. Each dot in the image represents the quality score of the corresponding position along reads.


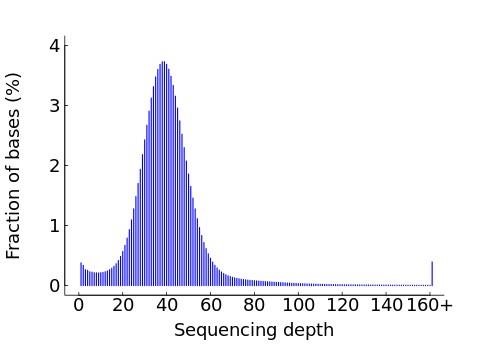


**Supplementary Figure 7 |** The distribution of per-base sequencing depth on the genome (SID-E2). X-axis denotes sequencing depth, while y-axis indicates the percentage of total genome under a given sequencing depth.


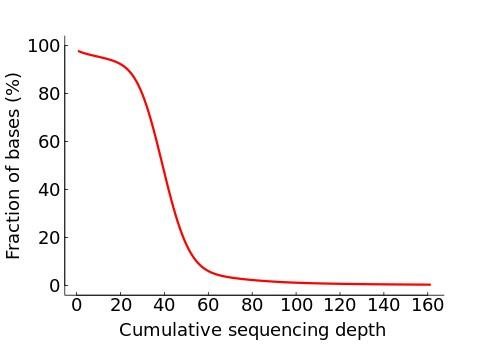


**Supplementary Figure 8 |** Cumulative depth distribution on the whole genome

(SIDE2). X-axis denotes sequencing depth, and Y-axis indicates the fraction of the whole genome excluding gap regions that achieves at or above a given sequencing depth.


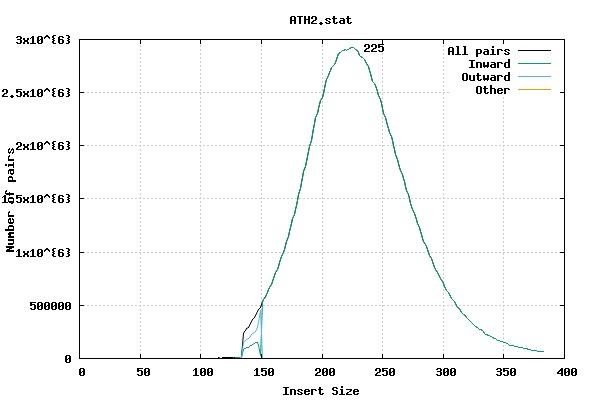


**Supplementary Figure 9 |** Insert size distribution of paired reads (SID-E2). X-axis denotes insert size of paired reads, and Y-axis shows the number of paired reads with a given insert size.


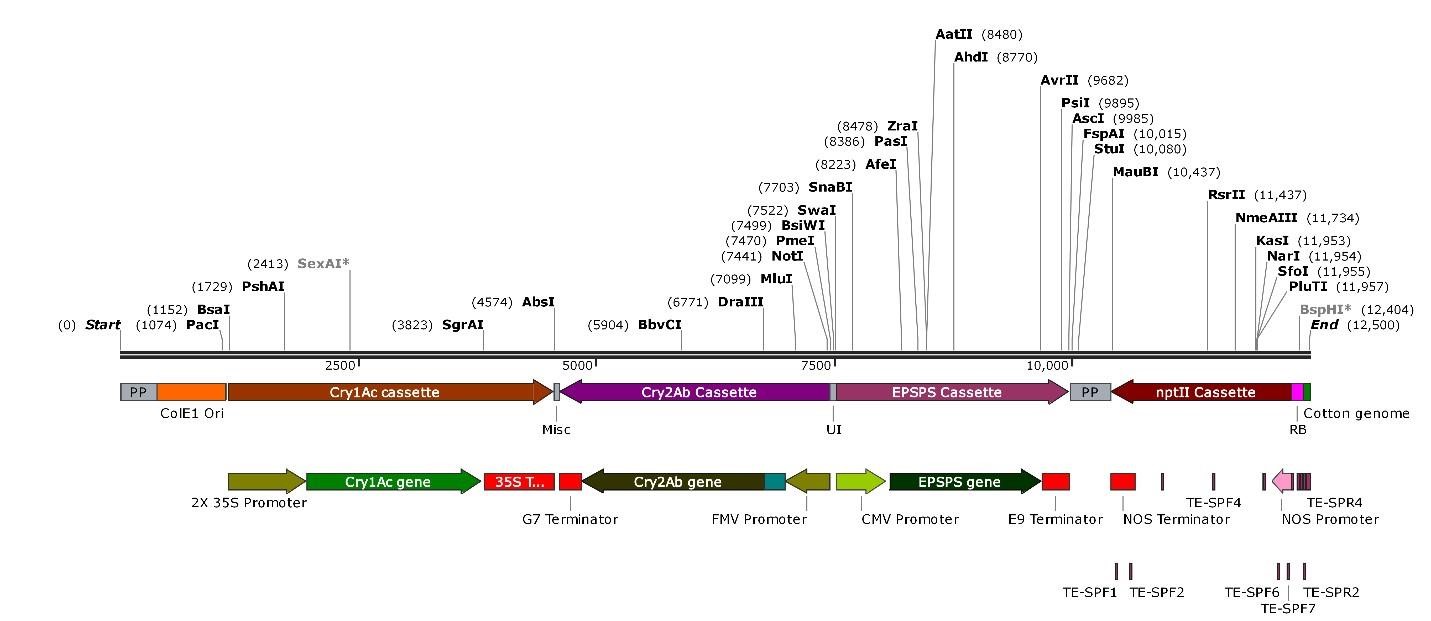


**Supplementary Figure 10 |** NIBGE 20-01 cotton specific event signature within cotton genome. Map showing different elements from triple gene NIBGE cotton. Sixty-two nucleotide bases of cotton genome were annotated near right border of T-DNA. The event specific primers were designed from cotton genome flanking T-DNA construct and from triple gene NIBGE cotton construct. These primer sequences were then used for the confirmation of triple gene NIBGE cotton event and was named as 20-01 event.


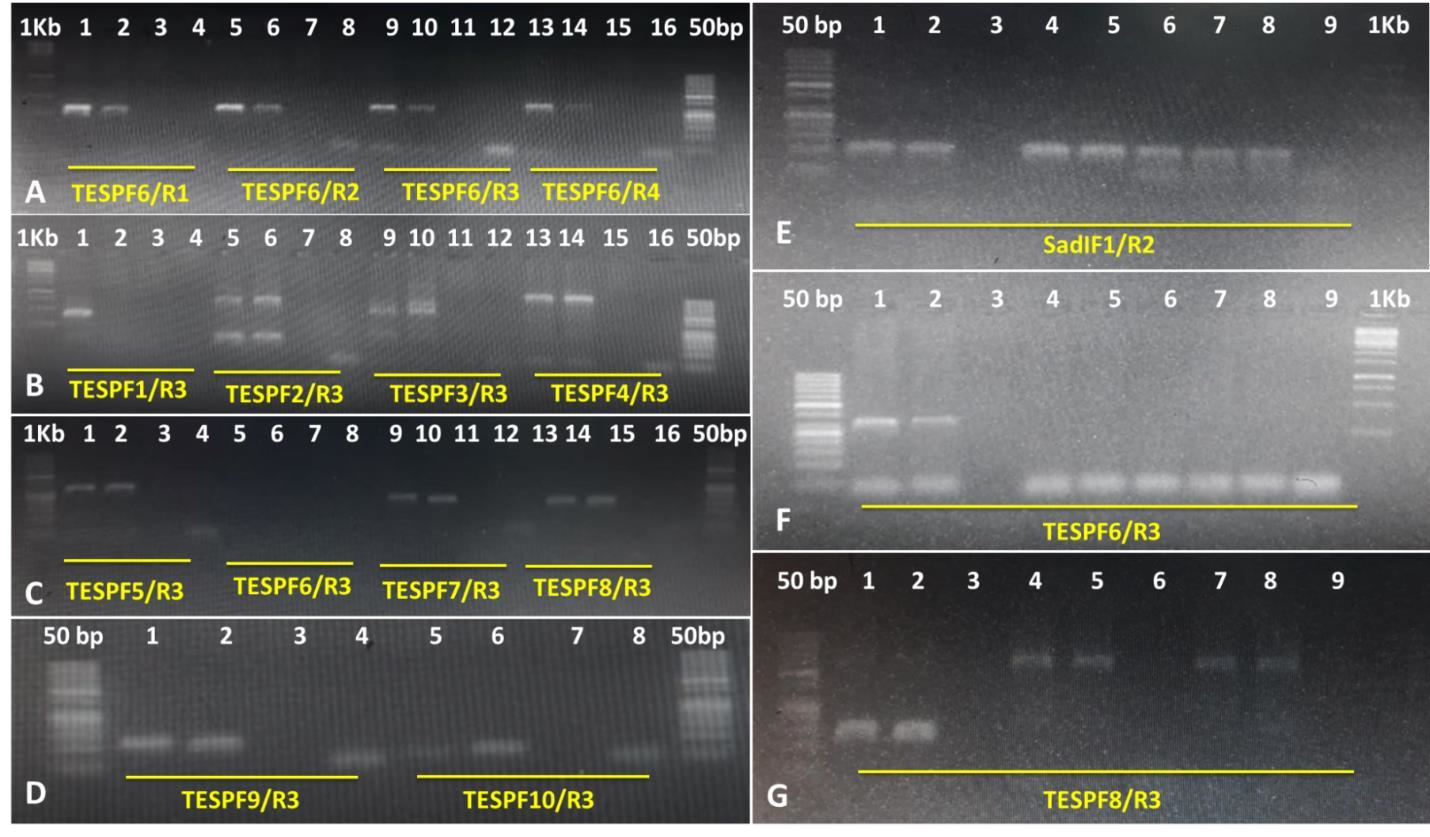


**Supplementary Figure 11 |** Validation of NGS-based characterized NIBGE triple gene cotton event through Event-specific PCR. A-C: 1-2, 5-6, 9-10, 13-14 = NIBGE triple gene cotton C3E2 P2-1 & P2-2 plants, respectively; 3, 7, 11, 15= non-GM coker control; 4,8,12,16= water control. **E-G**: 1= C3-E2-P2-1, 2= C3-E2-P2-2, 3= C3-E2-P2-20, 4= C2-E36-P1-4 (NIBGE double gene cotton), 5= non-GM coker cotton, 6= Mon531 cotton, 7= BGII cotton, 8= RR cotton, 9= water control. 50 bp= 50 bp DNA Marker, 1Kb= 1Kb DNA Marker. Yellow bars represent the respective PCR primer pairs used for amplification of samples.

**Additional Information**

Alignment of the complete cloned cassette found in the NIBGE-E2 cotton genome from NGS data, along with a complete cloned cassette original sequence has been shown as below;

NGS_cotton_genome GAACTTACTAAGCTATATGCTTACTCCCATCTCTTTTTCCATTTTCATATAGTATCACTA

Cloned_original_sequence GAACTTACTAAGCTATATGCTTACTCCCATCTCTTTTTCCATTTTCATATAGTATCACTA

************************************************************

NGS_cotton_genome AGCCGGATCAAGAGCTACCAACTCTTTTTCCGAAGGTAACTGGCTTCAGCAGAGCGCAGA

Cloned_original_sequence AGCCGGATCAAGAGCTACCAACTCTTTTTCCGAAGGTAACTGGCTTCAGCAGAGCGCAGA

************************************************************

NGS_cotton_genome TACCAAATACTGTCCTTCTAGTGTAGCCGTAGTTAGGCCACCACTTCAAGAACTCTGTAG

Cloned_original_sequence TACCAAATACTGTCCTTCTAGTGTAGCCGTAGTTAGGCCACCACTTCAAGAACTCTGTAG

************************************************************

NGS_cotton_genome CACCGCCTACATACCTCGCTCTGCTAATCCTGTTACCAGTGGCTGCTGCCAGTGGCGATA

Cloned_original_sequence CACCGCCTACATACCTCGCTCTGCTAATCCTGTTACCAGTGGCTGCTGCCAGTGGCGATA

************************************************************

NGS_cotton_genome AGTCGTGTCTTACCGGGTTGGACTCAAGACGATAGTTACCGGATAAGGCGCAGCGGTCGG

Cloned_original_sequence AGTCGTGTCTTACCGGGTTGGACTCAAGACGATAGTTACCGGATAAGGCGCAGCGGTCGG

************************************************************

NGS_cotton_genome GCTGAACGGGGGGTTCGTGCACACAGCCCAGCTTGGAGCGAACGACCTACACCGAACTGA

Cloned_original_sequence GCTGAACGGGGGGTTCGTGCACACAGCCCAGCTTGGAGCGAACGACCTACACCGAACTGA

************************************************************

NGS_cotton_genome GATACCTACAGCGTGAGCTATGAGAAAGCGCCACGCTTCCCGAAGGGAGAAAGGCGGACA

Cloned_original_sequence GATACCTACAGCGTGAGCTATGAGAAAGCGCCACGCTTCCCGAAGGGAGAAAGGCGGACA

************************************************************

NGS_cotton_genome GGTATCCGGTAAGCGGCAGGGTCGGAACAGGAGAGCGCACGAGGGAGCTTCCAGGGGGAA

Cloned_original_sequence GGTATCCGGTAAGCGGCAGGGTCGGAACAGGAGAGCGCACGAGGGAGCTTCCAGGGGGAA

************************************************************

NGS_cotton_genome ACGCCTGGTATCTTTATAGTCCTGTCGGGTTTCGCCACCTCTGACTTGAGCGTCGATTTT

Cloned_original_sequence ACGCCTGGTATCTTTATAGTCCTGTCGGGTTTCGCCACCTCTGACTTGAGCGTCGATTTT

************************************************************

NGS_cotton_genome TGTGATGCTCGTCAGGGGGGCGGAGCCTATGGAAAAACGCCAGCAACGCGGCCTTTTTAC

Cloned_original_sequence TGTGATGCTCGTCAGGGGGGCGGAGCCTATGGAAAAACGCCAGCAACGCGGCCTTTTTAC

************************************************************

NGS_cotton_genome GGTTCCTGGCCTTTTGCTGGCCTTTTGCTCACATGTTCTTTCCTGCGTTATCCCCTGATT

Cloned_original_sequence GGTTCCTGGCCTTTTGCTGGCCTTTTGCTCACATGTTCTTTCCTGCGTTATCCCCTGATT

************************************************************

NGS_cotton_genome CTGTGGATAACCGTATTACCGCCTTTGAGTGAGCTGATACCGCTCGCCGCAGCCGAACGA

Cloned_original_sequence CTGTGGATAACCGTATTACCGCCTTTGAGTGAGCTGATACCGCTCGCCGCAGCCGAACGA

************************************************************

NGS_cotton_genome CCGAGCGCAGCGAGTCAGTGAGCGAGGAAGCGGAAGAGCGCCTGATGCGGTATTTTCTCC

Cloned_original_sequence CCGAGCGCAGCGAGTCAGTGAGCGAGGAAGCGGAAGAGCGCCTGATGCGGTATTTTCTCC

************************************************************

NGS_cotton_genome TTACGCATCTGTGCGGTATTTCACACCGCATATGGTGCACTCTCAGTACAATCTGCTCTG

Cloned_original_sequence TTACGCATCTGTGCGGTATTTCACACCGCATATGGTGCACTCTCAGTACAATCTGCTCTG

************************************************************

NGS_cotton_genome ATGCCGCATAGTTAAGCCAGTATACACTCCGCTATCGCTACGTGACTGGGTCATGGCTGC

Cloned_original_sequence ATGCCGCATAGTTAAGCCAGTATACACTCCGCTATCGCTACGTGACTGGGTCATGGCTGC

************************************************************

NGS_cotton_genome GCCCCGACACCCGCCAACACCCGCTGACGCGCCCTGACGGGCTTGTCTGCTCCCGGCATC

Cloned_original_sequence GCCCCGACACCCGCCAACACCCGCTGACGCGCCCTGACGGGCTTGTCTGCTCCCGGCATC

************************************************************

NGS_cotton_genome CGCTTACAGACAAGCTGTGACCGTCTCCGGGAGCTGCATGTGTCAGAGGTTTTCACCGTC

Cloned_original_sequence CGCTTACAGACAAGCTGTGACCGTCTCCGGGAGCTGCATGTGTCAGAGGTTTTCACCGTC

************************************************************

NGS_cotton_genome ATCACCGAAACGCGCGAGGCAGCAGATCTATCGATGGTACCGTTAAATCTTAATTAATCC

Cloned_original_sequence ATCACCGAAACGCGCGAGGCAGCAGATCTATCGATGGTACCGTTAAATCTTAATTAATCC

************************************************************

NGS_cotton_genome CCAGATTAGCCTTCAATTTCAGAAAGAATGCTAACCCACAGATGGTTAGAGAGGCTTACG

Cloned_original_sequence CCAGATTAGCCTTCAATTTCAGAAAGAATGCTAACCCACAGATGGTTAGAGAGGCTTACG

************************************************************

NGS_cotton_genome CAGCAGGTCTCATCAAGACGATCTACCCGAGCAATAATCTCCAGGAAATCAAATACCTTC

Cloned_original_sequence CAGCAGGTCTCATCAAGACGATCTACCCGAGCAATAATCTCCAGGAAATCAAATACCTTC

************************************************************

NGS_cotton_genome CCAAGAAGGTTAAAGATGCAGTCAAAAGATTCAGGACTAACTGCATCAAGAACACAGAGA

Cloned_original_sequence CCAAGAAGGTTAAAGATGCAGTCAAAAGATTCAGGACTAACTGCATCAAGAACACAGAGA

************************************************************

NGS_cotton_genome AAGATATATTTCTCAAGATCAGAAGTACTATTCCAGTATGGACGATTCAAGGCTTGCTTC

Cloned_original_sequence AAGATATATTTCTCAAGATCAGAAGTACTATTCCAGTATGGACGATTCAAGGCTTGCTTC

************************************************************

NGS_cotton_genome ACAAACCAAGGCAAGTAATAGAGATTGGAGTCTCTAAAAAGGTAGTTCCCACTGAATCAA

Cloned_original_sequence ACAAACCAAGGCAAGTAATAGAGATTGGAGTCTCTAAAAAGGTAGTTCCCACTGAATCAA

************************************************************

NGS_cotton_genome AGGCCATGGAGTCAAAGATTCAAATAGAGGACCTAACAGAACTCGCCGTAAAGACTGGCG

Cloned_original_sequence AGGCCATGGAGTCAAAGATTCAAATAGAGGACCTAACAGAACTCGCCGTAAAGACTGGCG

************************************************************

NGS_cotton_genome AACAGTTCATACAGAGTCTCTTACGACTCAATGACAAGAAGAAAATCTTCGTCAACATGG

Cloned_original_sequence AACAGTTCATACAGAGTCTCTTACGACTCAATGACAAGAAGAAAATCTTCGTCAACATGG

************************************************************

NGS_cotton_genome TGGAGCACGACACACTTGTCTACTCCAAAAATATCAAAGATACAGTCTCAGAAGACCAAA

Cloned_original_sequence TGGAGCACGACACACTTGTCTACTCCAAAAATATCAAAGATACAGTCTCAGAAGACCAAA

************************************************************

NGS_cotton_genome GGGCAATTGAGACTTTTCAACAAAGGGTAATATCCGGAAACCTCCTCGGATTCCATTGCC

Cloned_original_sequence GGGCAATTGAGACTTTTCAACAAAGGGTAATATCCGGAAACCTCCTCGGATTCCATTGCC

************************************************************

NGS_cotton_genome CAGCTATCTGTCACTTTATTGTGAAGATAGTGGAAAAGGAAGGTGGCTCCTACAAATGCC

Cloned_original_sequence CAGCTATCTGTCACTTTATTGTGAAGATAGTGGAAAAGGAAGGTGGCTCCTACAAATGCC

************************************************************

NGS_cotton_genome ATCATTGCGATAAAGGAAAGGCCATCGTTGAAGATGCCTCTGCCGACAGTGGTCCCAAAG

Cloned_original_sequence ATCATTGCGATAAAGGAAAGGCCATCGTTGAAGATGCCTCTGCCGACAGTGGTCCCAAAG

************************************************************

NGS_cotton_genome ATGGACCCCCACCCACGAGGAGCATCGTGGAAAAAGAAGACGTTCCAACCACGTCTTCAA

Cloned_original_sequence ATGGACCCCCACCCACGAGGAGCATCGTGGAAAAAGAAGACGTTCCAACCACGTCTTCAA

************************************************************

NGS_cotton_genome AGCAAGTGGATTGATGTGATATCTCCACTGACGTAAGGGATGACGCACAATCCCACTATC

Cloned_original_sequence AGCAAGTGGATTGATGTGATATCTCCACTGACGTAAGGGATGACGCACAATCCCACTATC

************************************************************

NGS_cotton_genome CTTCGCAAGACCCTTCCTCTATATAAGGAAGTTCATTTCATTTGGAGAGACACGCTGACA

Cloned_original_sequence CTTCGCAAGACCCTTCCTCTATATAAGGAAGTTCATTTCATTTGGAGAGACACGCTGACA

************************************************************

NGS_cotton_genome AGCTGGATCCAAGCTCTAGGATCCAGCAGATCGAAATGGACAATAATCCGAATATTAATG

Cloned_original_sequence AGCTGGATCCAAGCTCTAGGATCCAGCAGATCGAAATGGACAATAATCCGAATATTAATG

************************************************************

NGS_cotton_genome AATGTATTCCCTACAATTGCCTCAGTAATCCAGAGGTCGAGGTCTTGGGAGGTGAAAGGA

Cloned_original_sequence AATGTATTCCCTACAATTGCCTCAGTAATCCAGAGGTCGAGGTCTTGGGAGGTGAAAGGA

************************************************************

NGS_cotton_genome TAGAAACCGGATATACCCCTATTGATATTAGCTTAAGTTTGACTCAATTCTTACTGTCCG

Cloned_original_sequence TAGAAACCGGATATACCCCTATTGATATTAGCTTAAGTTTGACTCAATTCTTACTGTCCG

************************************************************

NGS_cotton_genome AATTTGTACCTGGAGCAGGTTTCGTTCTTGGTTTGGTGGATATTATCTGGGGAATTTTCG

Cloned_original_sequence AATTTGTACCTGGAGCAGGTTTCGTTCTTGGTTTGGTGGATATTATCTGGGGAATTTTCG

************************************************************

NGS_cotton_genome GGCCAAGTCAGTGGGATGCTTTTCTGGTGCAGATTGAGCAACTTATTAACCAGCGTATTG

Cloned_original_sequence GGCCAAGTCAGTGGGATGCTTTTCTGGTGCAGATTGAGCAACTTATTAACCAGCGTATTG

************************************************************

NGS_cotton_genome AAGAATTTGCTAGAAATCAAGCTATTAGTCGATTGGAAGGATTATCCAACCTTTACCAAA

Cloned_original_sequence AAGAATTTGCTAGAAATCAAGCTATTAGTCGATTGGAAGGATTATCCAACCTTTACCAAA

************************************************************

NGS_cotton_genome TTTACGCTGAGAGCTTCCGAGAGTGGGAGGCTGATCCCACCAACCCAGCTTTGCGTGAAG

Cloned_original_sequence TTTACGCTGAGAGCTTCCGAGAGTGGGAGGCTGATCCCACCAACCCAGCTTTGCGTGAAG

************************************************************

NGS_cotton_genome AGATGAGGATTCAATTCAATGATATGAACAGCGCACTGACTACAGCTATACCTCTGTTTG

Cloned_original_sequence AGATGAGGATTCAATTCAATGATATGAACAGCGCACTGACTACAGCTATACCTCTGTTTG

************************************************************

NGS_cotton_genome CTGTGCAAAACTACCAGGTGCCTCTCCTCTCCGTTTACGTTCAGGCTGCAAATCTTCATC

Cloned_original_sequence CTGTGCAAAACTACCAGGTGCCTCTCCTCTCCGTTTACGTTCAGGCTGCAAATCTTCATC

************************************************************

NGS_cotton_genome TCAGTGTTCTTAGAGACGTTTCTGTTTTTGGGCAGCGTTGGGGCTTCGATGCTGCAACTA

Cloned_original_sequence TCAGTGTTCTTAGAGACGTTTCTGTTTTTGGGCAGCGTTGGGGCTTCGATGCTGCAACTA

************************************************************

NGS_cotton_genome TAAACTCTCGTTACAACGATCTCACTCGTCTTATTGGCAACTACACAGATTACGCAGTAA

Cloned_original_sequence TAAACTCTCGTTACAACGATCTCACTCGTCTTATTGGCAACTACACAGATTACGCAGTAA

************************************************************

NGS_cotton_genome GGTGGTATAACACAGGCTTGGAGAGAGTCTGGGGACCGGACTCTCGAGATTGGGTGAGGT

Cloned_original_sequence GGTGGTATAACACAGGCTTGGAGAGAGTCTGGGGACCGGACTCTCGAGATTGGGTGAGGT

************************************************************

NGS_cotton_genome ATAACCAATTTAGGAGAGAGCTTACCCTTACTGTTCTCGATATAGTGGCTCTTTTCCCTA

Cloned_original_sequence ATAACCAATTTAGGAGAGAGCTTACCCTTACTGTTCTCGATATAGTGGCTCTTTTCCCTA

************************************************************

NGS_cotton_genome ATTACGATTCCAGAAGATATCCCATTAGGACCGTGAGCCAACTGACTAGGGAAATCTATA

Cloned_original_sequence ATTACGATTCCAGAAGATATCCCATTAGGACCGTGAGCCAACTGACTAGGGAAATCTATA

************************************************************

NGS_cotton_genome CAAACCCAGTTCTCGAAAACTTCGACGGTTCTTTTAGAGGTAGTGCCCAAGGTATAGAAA

Cloned_original_sequence CAAACCCAGTTCTCGAAAACTTCGACGGTTCTTTTAGAGGTAGTGCCCAAGGTATAGAAA

************************************************************

NGS_cotton_genome GATCTATTAGGTCACCTCACCTTATGGACATACTCAATAGCATTACAATTTATACAGACG

Cloned_original_sequence GATCTATTAGGTCACCTCACCTTATGGACATACTCAATAGCATTACAATTTATACAGACG

************************************************************

NGS_cotton_genome CCCATAGGGGCTATTATTATTGGAGCGGTCACCAGATCATGGCTAGTCCAGTCGGCTTCT

Cloned_original_sequence CCCATAGGGGCTATTATTATTGGAGCGGTCACCAGATCATGGCTAGTCCAGTCGGCTTCT

************************************************************

NGS_cotton_genome CAGGACCGGAGTTTACCTTTCCTCTGTACGGGACTATGGGTAACGCAGCTCCCCAGCAAA

Cloned_original_sequence CAGGACCGGAGTTTACCTTTCCTCTGTACGGGACTATGGGTAACGCAGCTCCCCAGCAAA

************************************************************

NGS_cotton_genome GAATCGTGGCCCAGCTCGGTCAGGGGGTGTATAGGACCCTCTCATCTACCTTGTACAGAA

Cloned_original_sequence GAATCGTGGCCCAGCTCGGTCAGGGGGTGTATAGGACCCTCTCATCTACCTTGTACAGAA

************************************************************

NGS_cotton_genome GACCGTTCAATATCGGCATAAATAACCAACAATTGAGCGTTTTAGACGGTACAGAATTCG

Cloned_original_sequence GACCGTTCAATATCGGCATAAATAACCAACAATTGAGCGTTTTAGACGGTACAGAATTCG

************************************************************

NGS_cotton_genome CTTATGGTACTAGCTCTAATTTACCCTCCGCAGTATACCGAAAGTCCGGAACCGTAGATT

Cloned_original_sequence CTTATGGTACTAGCTCTAATTTACCCTCCGCAGTATACCGAAAGTCCGGAACCGTAGATT

************************************************************

NGS_cotton_genome CATTGGACGAGATCCCACCACAAAACAATAATGTGCCTCCACGTCAAGGATTTTCCCATA

Cloned_original_sequence CATTGGACGAGATCCCACCACAAAACAATAATGTGCCTCCACGTCAAGGATTTTCCCATA

************************************************************

NGS_cotton_genome GATTGTCACATGTTTCAATGTTTAGGTCCGGGTTTTCTAACTCCAGTGTTAGTATCATAA

Cloned_original_sequence GATTGTCACATGTTTCAATGTTTAGGTCCGGGTTTTCTAACTCCAGTGTTAGTATCATAA

************************************************************

NGS_cotton_genome GAGCTCCTATGTTCTCTTGGATCCACCGTTCAGCCGAATTTAACAACATAATTGCTTCTG

Cloned_original_sequence GAGCTCCTATGTTCTCTTGGATCCACCGTTCAGCCGAATTTAACAACATAATTGCTTCTG

************************************************************

NGS_cotton_genome ATTCAATCACTCAAATTCCCGCTGTCAAAGGCAATTTCTTGTTCAATGGTAGCGTAATTT

Cloned_original_sequence ATTCAATCACTCAAATTCCCGCTGTCAAAGGCAATTTCTTGTTCAATGGTAGCGTAATTT

************************************************************

NGS_cotton_genome CTGGGCCTGGGTTTACAGGTGGGGATCTTGTCAGGTTGAACAGCTCTGGAAACAACATCC

Cloned_original_sequence CTGGGCCTGGGTTTACAGGTGGGGATCTTGTCAGGTTGAACAGCTCTGGAAACAACATCC

************************************************************

NGS_cotton_genome AGAATAGGGGATACATCGAAGTCCCTATCCATTTTCCATCCACTTCTACTAGATATAGGG

Cloned_original_sequence AGAATAGGGGATACATCGAAGTCCCTATCCATTTTCCATCCACTTCTACTAGATATAGGG

************************************************************

NGS_cotton_genome TTAGGGTCAGATATGCCTCAGTTACACCAATTCATCTGAACGTTAATTGGGGAAATTCAT

Cloned_original_sequence TTAGGGTCAGATATGCCTCAGTTACACCAATTCATCTGAACGTTAATTGGGGAAATTCAT

************************************************************

NGS_cotton_genome CAATCTTCTCTAACACTGTGCCTGCCACCGCAACTTCCCTCGATAACCTTCAAAGTTCAG

Cloned_original_sequence CAATCTTCTCTAACACTGTGCCTGCCACCGCAACTTCCCTCGATAACCTTCAAAGTTCAG

************************************************************

NGS_cotton_genome ATTTCGGTTATTTTGAGAGTGCCAATGCTTTCACATCTAGCCTTGGTAATATCGTTGGAG

Cloned_original_sequence ATTTCGGTTATTTTGAGAGTGCCAATGCTTTCACATCTAGCCTTGGTAATATCGTTGGAG

************************************************************

NGS_cotton_genome TTCGAAATTTCTCCGGAACTGCCGGCGTTATCATTGATAGATTTGAGTTCATCCCAGTTA

Cloned_original_sequence TTCGAAATTTCTCCGGAACTGCCGGCGTTATCATTGATAGATTTGAGTTCATCCCAGTTA

************************************************************

NGS_cotton_genome CTGCAACTTTAGAATGATCTAGAAGCTTAGCAGCTTACCGTCACCGGTGTGAGGGAACTA

Cloned_original_sequence CTGCAACTTTAGAATGATCTAGAAGCTTAGCAGCTTACCGTCACCGGTGTGAGGGAACTA

************************************************************

NGS_cotton_genome GTTTTGATCTTGAAAGATCTTTTATCTTTAGAGTTAAGAACTCTTTCGTATTTTGGTGAG

Cloned_original_sequence GTTTTGATCTTGAAAGATCTTTTATCTTTAGAGTTAAGAACTCTTTCGTATTTTGGTGAG

************************************************************

NGS_cotton_genome GTTTTATCCTCTTGAGTTTTGGTCATAGACCTATTCATGGCTCTGATACCAATTTTTAAG

Cloned_original_sequence GTTTTATCCTCTTGAGTTTTGGTCATAGACCTATTCATGGCTCTGATACCAATTTTTAAG

************************************************************

NGS_cotton_genome CGGGGGCTTATGCGGATTATTTCTTAAATTGATAAGGGGTTATTAGGGGGTATAGGGTAT

Cloned_original_sequence CGGGGGCTTATGCGGATTATTTCTTAAATTGATAAGGGGTTATTAGGGGGTATAGGGTAT

************************************************************

NGS_cotton_genome AAATACAAGCATTCCCTTAGCGTATAGTATAAGTATAGTAGCGTACCTCTATCAAATTTC

Cloned_original_sequence AAATACAAGCATTCCCTTAGCGTATAGTATAAGTATAGTAGCGTACCTCTATCAAATTTC

************************************************************

NGS_cotton_genome CATCTTCTTACCTTGCACAGGGCCTGCAACCTTATCCTTCCTTGTCTTCCTCCTTCCTTC

Cloned_original_sequence CATCTTCTTACCTTGCACAGGGCCTGCAACCTTATCCTTCCTTGTCTTCCTCCTTCCTTC

************************************************************

NGS_cotton_genome CGTCCACTTCATCATATTTAAACCAAACCTACGGGGGAGTCAACGTAACCAACCCTGCCT

Cloned_original_sequence CGTCCACTTCATCATATTTAAACCAAACCTACGGGGGAGTCAACGTAACCAACCCTGCCT

************************************************************

NGS_cotton_genome TAGCATCTTTTCCCTAACGGCCTCCTGCCTAAGCGGTACTTCTAGCTTCGAACGGCGTCT

Cloned_original_sequence TAGCATCTTTTCCCTAACGGCCTCCTGCCTAAGCGGTACTTCTAGCTTCGAACGGCGTCT

************************************************************

NGS_cotton_genome GGGCTCCAGGTTTAGTCGTCTCGTGTCTGGTTTATATTCACGACAAAGATCTATAGGGAC

Cloned_original_sequence GGGCTCCAGGTTTAGTCGTCTCGTGTCTGGTTTATATTCACGACAAAGATCTATAGGGAC

************************************************************

NGS_cotton_genome TTTAGGAGATCTGGATTTTAGTACTGGATTTTGGTTTTAGGAATTAGAAATTTTATTGAT

Cloned_original_sequence TTTAGGAGATCTGGATTTTAGTACTGGATTTTGGTTTTAGGAATTAGAAATTTTATTGAT

************************************************************

NGS_cotton_genome AGAAGTATTTTACAAATACAAATACATACTAAGGGTTTCTTATATGCTCAACACATGAGC

Cloned_original_sequence AGAAGTATTTTACAAATACAAATACATACTAAGGGTTTCTTATATGCTCAACACATGAGC

************************************************************

NGS_cotton_genome GAAACCCTATAAGAACCCTAATTTCCCTTATCGGGAAACTACTCACACATTATTTATGGA

Cloned_original_sequence GAAACCCTATAAGAACCCTAATTTCCCTTATCGGGAAACTACTCACACATTATTTATGGA

************************************************************

NGS_cotton_genome GAAAATAGAGAGAGATAGATTTGTAGAGAGAGACTGGTGATTTCAGCGTACCGTCCGAAC

Cloned_original_sequence GAAAATAGAGAGAGATAGATTTGTAGAGAGAGACTGGTGATTTCAGCGTACCGTCCGAAC

************************************************************

NGS_cotton_genome CTTAGGTGTCGACCTCGAGGGGGGGCCCGGTACCGGGCCCAAGATCTGGCCCATAAGCTT

Cloned_original_sequence CTTAGGTGTCGACCTCGAGGGGGGGCCCGGTACCGGGCCCAAGATCTGGCCCATAAGCTT

************************************************************

NGS_cotton_genome TACACGTCGACGATCTTGAAAGAAATATAGTTTAAATATTTATTGATAAAATAACAAGTC

Cloned_original_sequence TACACGTCGACGATCTTGAAAGAAATATAGTTTAAATATTTATTGATAAAATAACAAGTC

************************************************************

NGS_cotton_genome AGGTATTATAGTCCAAGCAAAAACATAAATTTATTGATGCAAGTTTAAATTCAGAAATAT

Cloned_original_sequence AGGTATTATAGTCCAAGCAAAAACATAAATTTATTGATGCAAGTTTAAATTCAGAAATAT

************************************************************

NGS_cotton_genome TTCAATAACTGATTATATCAGCTGGTACATTGCCGTAGATGAAAGACTGAGTGCGATATT

Cloned_original_sequence TTCAATAACTGATTATATCAGCTGGTACATTGCCGTAGATGAAAGACTGAGTGCGATATT

************************************************************

NGS_cotton_genome ATGTGTAATACATAAATTGATGATATAGCTAGCTTAGCTCATCGGTCGACTCAATAAAGC

Cloned_original_sequence ATGTGTAATACATAAATTGATGATATAGCTAGCTTAGCTCATCGGTCGACTCAATAAAGC

************************************************************

NGS_cotton_genome GGGGAAATATTGGTTGGCACCAGCATAATGTTCATCAAGTCGAATTGTGTCCCTGAATTA

Cloned_original_sequence GGGGAAATATTGGTTGGCACCAGCATAATGTTCATCAAGTCGAATTGTGTCCCTGAATTA

************************************************************

NGS_cotton_genome AGGGTCACATTGATGTCAAGTGGCACGTCGCTATTTGAGGAAGCAACTACGTTTCCTATG

Cloned_original_sequence AGGGTCACATTGATGTCAAGTGGCACGTCGCTATTTGAGGAAGCAACTACGTTTCCTATG

************************************************************

NGS_cotton_genome TTAATATCTGAAAATCGTGCTCCATTGTCGTTGACTCCATCGTTGTTGGTAGTAGTGTTG

Cloned_original_sequence TTAATATCTGAAAATCGTGCTCCATTGTCGTTGACTCCATCGTTGTTGGTAGTAGTGTTG

************************************************************

NGS_cotton_genome ACATTAGTTGCTGTATAGACTCTCCCGTTGATGGTAACCCTTATTGTAGAGTTTCCGATT

Cloned_original_sequence ACATTAGTTGCTGTATAGACTCTCCCGTTGATGGTAACCCTTATTGTAGAGTTTCCGATT

************************************************************

NGS_cotton_genome GAAGAGACTCGCAGATAGAGGTTGTAACTATTTCCATTACCTCTTAAGGTATATCTGGCG

Cloned_original_sequence GAAGAGACTCGCAGATAGAGGTTGTAACTATTTCCATTACCTCTTAAGGTATATCTGGCG

************************************************************

NGS_cotton_genome GTGGTGTTATTTTGCTCGAACCTGAGAGAGTCTCCTTGGTTCCCAAATTTTTCGGAAATG

Cloned_original_sequence GTGGTGTTATTTTGCTCGAACCTGAGAGAGTCTCCTTGGTTCCCAAATTTTTCGGAAATG

************************************************************

NGS_cotton_genome AATGTTCTTGTTTGGTTATTAACTTGAGTGGCGTGTATGGGGCTAATTGTGAAACCGGTG

Cloned_original_sequence AATGTTCTTGTTTGGTTATTAACTTGAGTGGCGTGTATGGGGCTAATTGTGAAACCGGTG

************************************************************

NGS_cotton_genome TAATCATTTGGGGCCAAATGAATCATAGAACCGTTTTCGTGCACAGCATGAATATTGTTT

Cloned_original_sequence TAATCATTTGGGGCCAAATGAATCATAGAACCGTTTTCGTGCACAGCATGAATATTGTTT

************************************************************

NGS_cotton_genome TTCCTGTTATGAACTGAAACCATGTAAGCACGAGCACCACCAGGGGTTCCACTTGGGGAA

Cloned_original_sequence TTCCTGTTATGAACTGAAACCATGTAAGCACGAGCACCACCAGGGGTTCCACTTGGGGAA

************************************************************

NGS_cotton_genome GCAATGTTCCTAATCTCATTGTAATGGAGCGGTCGCCTCAAATCCTCGTTTCTGACTACA

Cloned_original_sequence GCAATGTTCCTAATCTCATTGTAATGGAGCGGTCGCCTCAAATCCTCGTTTCTGACTACA

************************************************************

NGS_cotton_genome AGTGGGACTCCGGAGATGTTTCTTATGAAGTAATCAGGGAAGTAATTTGAGTTCCCCCTT

Cloned_original_sequence AGTGGGACTCCGGAGATGTTTCTTATGAAGTAATCAGGGAAGTAATTTGAGTTCCCCCTT

************************************************************

NGS_cotton_genome GCAGTGAAGGCTCCTGAACGCAAACCAAGAGTGGTTTCGAAGCTCTCAGTTTGCCAATTT

Cloned_original_sequence GCAGTGAAGGCTCCTGAACGCAAACCAAGAGTGGTTTCGAAGCTCTCAGTTTGCCAATTT

************************************************************

NGS_cotton_genome GTAACTGTAGCCACGCCTTCCCTATCGCTGCCGGAGTCGAGCCAGGAACGAACGAAGGGG

Cloned_original_sequence GTAACTGTAGCCACGCCTTCCCTATCGCTGCCGGAGTCGAGCCAGGAACGAACGAAGGGG

************************************************************

NGS_cotton_genome GTCAACAGAGGTGGAAGAAATGTTGAACAATTGAAGTTCTGGTTGAATGGAGAAGCCCCA

Cloned_original_sequence GTCAACAGAGGTGGAAGAAATGTTGAACAATTGAAGTTCTGGTTGAATGGAGAAGCCCCA

************************************************************

NGS_cotton_genome ATATCGCCTGAAGAGATACCTCCACTGTAATTAACCCTAGCTGCTAAAAGTGCATGAGTT

Cloned_original_sequence ATATCGCCTGAAGAGATACCTCCACTGTAATTAACCCTAGCTGCTAAAAGTGCATGAGTT

************************************************************

NGS_cotton_genome GTGGTAGAACCGGGCAGACCGACGATATTAGGGAAAGTGTTTGATAATCGAGCACCAGAA

Cloned_original_sequence GTGGTAGAACCGGGCAGACCGACGATATTAGGGAAAGTGTTTGATAATCGAGCACCAGAA

************************************************************

NGS_cotton_genome AAGCCATTAAGCACGTAGTTACTATTCACTTGAAAAAGGGAGTATAAGAATGGCCAATCC

Cloned_original_sequence AAGCCATTAAGCACGTAGTTACTATTCACTTGAAAAAGGGAGTATAAGAATGGCCAATCC

************************************************************

NGS_cotton_genome TGGGAAGTAAAGGATTGTGTCTGCTGAGGACCGCTACCACTGGCATACAAGTTAGCACCG

Cloned_original_sequence TGGGAAGTAAAGGATTGTGTCTGCTGAGGACCGCTACCACTGGCATACAAGTTAGCACCG

************************************************************

NGS_cotton_genome CTGCTAACCAACAAAGATTGATACTTAAACAAACTCCAGATACTAACGTACTCAAACACA

Cloned_original_sequence CTGCTAACCAACAAAGATTGATACTTAAACAAACTCCAGATACTAACGTACTCAAACACA

************************************************************

NGS_cotton_genome TTCAGGAACATGTAAGTCCTAAATTCAAGCATGTCGTGAAGACGGGTATTAAGACCTTTG

Cloned_original_sequence TTCAGGAACATGTAAGTCCTAAATTCAAGCATGTCGTGAAGACGGGTATTAAGACCTTTG

************************************************************

NGS_cotton_genome AATGCACTCTGATAGGTGTTTATGCAATAGTTGGAATAGTCACGTGTGTAGTTCTTAAGA

Cloned_original_sequence AATGCACTCTGATAGGTGTTTATGCAATAGTTGGAATAGTCACGTGTGTAGTTCTTAAGA

************************************************************

NGS_cotton_genome TAATCACGATATGTACGCAGGGTGGCTGCTGAAATACCCCACTCATCAGCATTAAGTATG

Cloned_original_sequence TAATCACGATATGTACGCAGGGTGGCTGCTGAAATACCCCACTCATCAGCATTAAGTATG

************************************************************

NGS_cotton_genome ACATCCCTGATGAATGAAAGATGCAAGTTTGCTGCCTGGGCAAAAAGAGGGAGTAAAAGC

Cloned_original_sequence ACATCCCTGATGAATGAAAGATGCAAGTTTGCTGCCTGGGCAAAAAGAGGGAGTAAAAGC

************************************************************

NGS_cotton_genome AGTTGATACCCCTGCATTTGAAATTGGGGCAACCTGTTCAGAAATAATTGTTGCATAGTA

Cloned_original_sequence AGTTGATACCCCTGCATTTGAAATTGGGGCAACCTGTTCAGAAATAATTGTTGCATAGTA

************************************************************

NGS_cotton_genome TTTACGCTGGATGTGATACTCAGAGGTACAGCGTTACGGTTCGGGTTTAAGAAATTATCA

Cloned_original_sequence TTTACGCTGGATGTGATACTCAGAGGTACAGCGTTACGGTTCGGGTTTAAGAAATTATCA

************************************************************

NGS_cotton_genome ACCTGTCTATTAAACTCTTCCACATTAGCCTGCAATCCTGTGAGTTCAGCATTCACTCTA

Cloned_original_sequence ACCTGTCTATTAAACTCTTCCACATTAGCCTGCAATCCTGTGAGTTCAGCATTCACTCTA

************************************************************

NGS_cotton_genome GCTAAAGTGTCAGTGTTCAGACGCTGATTAAGGAATTTCTCAGTCTCTCTGAGGATATCT

Cloned_original_sequence GCTAAAGTGTCAGTGTTCAGACGCTGATTAAGGAATTTCTCAGTCTCTCTGAGGATATCT

************************************************************

NGS_cotton_genome TGCATGAGATTTGTGGATCCAGAAGGAAAGATCAAATTTCTGAGTTCGGAAAGAATCCTC

Cloned_original_sequence TGCATGAGATTTGTGGATCCAGAAGGAAAGATCAAATTTCTGAGTTCGGAAAGAATCCTC

************************************************************

NGS_cotton_genome TTTCCAACCAAGCTGCCAACTTTCTTCAAGAGAAATGATGCTACAGTGCCAACAATAGGA

Cloned_original_sequence TTTCCAACCAAGCTGCCAACTTTCTTCAAGAGAAATGATGCTACAGTGCCAACAATAGGA

************************************************************

NGS_cotton_genome TCGAGATACAGGCTATGGTTATTCTTCTTCCATTCGGTCCATTCTTTTTGAACAGTATCC

Cloned_original_sequence TCGAGATACAGGCTATGGTTATTCTTCTTCCATTCGGTCCATTCTTTTTGAACAGTATCC

************************************************************

NGS_cotton_genome AAAGACTTGTGCTGAAAGCTAAAAGGATCGTGAGCAGCAACATTATAGGCATCACAAATT

Cloned_original_sequence AAAGACTTGTGCTGAAAGCTAAAAGGATCGTGAGCAGCAACATTATAGGCATCACAAATT

************************************************************

NGS_cotton_genome GTAGTTCTCCCAGAATTAAGCACAGAATTGTCCATGGCAAGCATGCACGCCGTGGAAACA

Cloned_original_sequence GTAGTTCTCCCAGAATTAAGCACAGAATTGTCCATGGCAAGCATGCACGCCGTGGAAACA

************************************************************

NGS_cotton_genome GAAGACATGACCTTAAGAGGACGAAGCTCAGAGCCAATTAACGTCATCCCACTCTTCTTC

Cloned_original_sequence GAAGACATGACCTTAAGAGGACGAAGCTCAGAGCCAATTAACGTCATCCCACTCTTCTTC

************************************************************

NGS_cotton_genome AATCCCCACGACGACGAAATCGGATAAGCTCGTGGATGCTGCTGCGTCTTCAGAGAAACC

Cloned_original_sequence AATCCCCACGACGACGAAATCGGATAAGCTCGTGGATGCTGCTGCGTCTTCAGAGAAACC

************************************************************

NGS_cotton_genome GATAAGGGAGATTTGCGTTGACTGGATTTCGAGAGATTGGAGATAAGAGATGGGTTCTGC

Cloned_original_sequence GATAAGGGAGATTTGCGTTGACTGGATTTCGAGAGATTGGAGATAAGAGATGGGTTCTGC

************************************************************

NGS_cotton_genome ACACCATTGCAGATTCTGCTAACTTGCGCCATTGTACCGGTATCGATATTGGTTGAGTAT

Cloned_original_sequence ACACCATTGCAGATTCTGCTAACTTGCGCCATTGTACCGGTATCGATATTGGTTGAGTAT

************************************************************

NGS_cotton_genome TGATGATCTTCAAATGGGAATGAATCCGTCTTTAAATAGATGGAATTCTTGAGTGGATGT

Cloned_original_sequence TGATGATCTTCAAATGGGAATGAATCCGTCTTTAAATAGATGGAATTCTTGAGTGGATGT

************************************************************

NGS_cotton_genome CGTCACTGCGTTCGTAATACGCGTTTGTGAGTGGGCTTCCACATGGGCTGCTTTGCAAGC

Cloned_original_sequence CGTCACTGCGTTCGTAATACGCGTTTGTGAGTGGGCTTCCACATGGGCTGCTTTGCAAGC

************************************************************

NGS_cotton_genome TGTCCTCCCCATTATTGTACCGGCTGTCTGCCATGATACTTTTATCACGTGGATGGGTTC

Cloned_original_sequence TGTCCTCCCCATTATTGTACCGGCTGTCTGCCATGATACTTTTATCACGTGGATGGGTTC

************************************************************

NGS_cotton_genome CACTTTGAGGTGACTTGTCTGCCACTTCTTTGTTGTCACGTGGGTGGTCCCCACTGCAGT

Cloned_original_sequence CACTTTGAGGTGACTTGTCTGCCACTTCTTTGTTGTCACGTGGGTGGTCCCCACTGCAGT

************************************************************

NGS_cotton_genome ACTGGCAAAGATGCTCTTCGTACTTTTTCTGTAGAATTTTACACCACAATTTCTTTGTGT

Cloned_original_sequence ACTGGCAAAGATGCTCTTCGTACTTTTTCTGTAGAATTTTACACCACAATTTCTTTGTGT

************************************************************

NGS_cotton_genome GGTCCCCACAATTGATTTGATTGATGTTGATCAATCTTTTCTCAAAAAGTTTTACCTTGT

Cloned_original_sequence GGTCCCCACAATTGATTTGATTGATGTTGATCAATCTTTTCTCAAAAAGTTTTACCTTGT

************************************************************

NGS_cotton_genome GAATTTCTTCCGTGGTAATCTTTTTTGTACACTTGCTCGAGCGTCGACGTACTAACAGTG

Cloned_original_sequence GAATTTCTTCCGTGGTAATCTTTTTTGTACACTTGCTCGAGCGTCGACGTACTAACAGTG

************************************************************

NGS_cotton_genome CGGCCGCAATCTCCAGGACCGAGGGTGTTTAAACACTAATTTAACGAGCTCTAGAACTCG

Cloned_original_sequence CGGCCGCAATCTCCAGGACCGAGGGTGTTTAAACACTAATTTAACGAGCTCTAGAACTCG

************************************************************

NGS_cotton_genome TACGTCAGGTACCCGCGGATTTAAATGAAGGTAATTATCCAAGATGTAGCATCAAGAATC

Cloned_original_sequence TACGTCAGGTACCCGCGGATTTAAATGAAGGTAATTATCCAAGATGTAGCATCAAGAATC

************************************************************

NGS_cotton_genome CAATGTTTACGGGAAAAACTATGGAAGTATTATGTGAGCTCAGCAAGAAGCAGATCAATA

Cloned_original_sequence CAATGTTTACGGGAAAAACTATGGAAGTATTATGTGAGCTCAGCAAGAAGCAGATCAATA

************************************************************

NGS_cotton_genome TGCGGCACATATGCAACCTATGTTCAAAAATGAAGAATGTACAGATACAAGATCCTATAC

Cloned_original_sequence TGCGGCACATATGCAACCTATGTTCAAAAATGAAGAATGTACAGATACAAGATCCTATAC

************************************************************

NGS_cotton_genome TGCCAGAATACGAAGAAGAATACGTAGAAATTGAAAAAGAAGAACCAGGCGAAGAAAAGA

Cloned_original_sequence TGCCAGAATACGAAGAAGAATACGTAGAAATTGAAAAAGAAGAACCAGGCGAAGAAAAGA

************************************************************

NGS_cotton_genome ATCTTGAAGACGTAAGCACTGACGACAACAATGAAAAGAAGAAGATAAGGTCGGTGATTG

Cloned_original_sequence ATCTTGAAGACGTAAGCACTGACGACAACAATGAAAAGAAGAAGATAAGGTCGGTGATTG

************************************************************

NGS_cotton_genome TGAAAGAGACATAGAGGACACATGTAAGGTGGAAAATGTAAGGGCGGAAAGTAACCTTAT

Cloned_original_sequence TGAAAGAGACATAGAGGACACATGTAAGGTGGAAAATGTAAGGGCGGAAAGTAACCTTAT

************************************************************

NGS_cotton_genome CACAAAGGAATCTTATCCCCCACTACTTATCCTTTTATATTTTTCCGTGTCATTTTTGCC

Cloned_original_sequence CACAAAGGAATCTTATCCCCCACTACTTATCCTTTTATATTTTTCCGTGTCATTTTTGCC

************************************************************

NGS_cotton_genome CTTGAGTTTTCCTATATAAGGAACCAAGTTCGGCATTTGTGAAAACAAGAAAAAATTTGG

Cloned_original_sequence CTTGAGTTTTCCTATATAAGGAACCAAGTTCGGCATTTGTGAAAACAAGAAAAAATTTGG

************************************************************

NGS_cotton_genome TGTAAGCTATTTTCTTTGAAGTACTGAGGATACAACTTCAGAGAAATTTGTAAGTTTGTG

Cloned_original_sequence TGTAAGCTATTTTCTTTGAAGTACTGAGGATACAACTTCAGAGAAATTTGTAAGTTTGTG

************************************************************

NGS_cotton_genome GTGAAGGATCCGAACCTGAATTCCAGCACACTGGCGGCCGTTACTAGTGGATCCGAAATG

Cloned_original_sequence GTGAAGGATCCGAACCTGAATTCCAGCACACTGGCGGCCGTTACTAGTGGATCCGAAATG

************************************************************

NGS_cotton_genome GCTCAAATAAACAACATGGCTCAAGGCATCCAGACTCTTAACCCAAACTCCAACTTCCAT

Cloned_original_sequence GCTCAAATAAACAACATGGCTCAAGGCATCCAGACTCTTAACCCAAACTCCAACTTCCAT

************************************************************

NGS_cotton_genome AAGCCTCAAGTGCCTAAATCCTCATCCTTTTTAGTTTTCGGAAGCAAGAAACTTAAGAAT

Cloned_original_sequence AAGCCTCAAGTGCCTAAATCCTCATCCTTTTTAGTTTTCGGAAGCAAGAAACTTAAGAAT

************************************************************

NGS_cotton_genome AGCGCTAATAGCATGCTTGTTCTTAAAAAGGATTCCATTTTTATGCAAAAATTCTGCAGT

Cloned_original_sequence AGCGCTAATAGCATGCTTGTTCTTAAAAAGGATTCCATTTTTATGCAAAAATTCTGCAGT

************************************************************

NGS_cotton_genome TTTAGAATATCAGCATCCGTCGCTACCGCCTGTATGCTCCATGGGGCTAGTTCTAGGCCA

Cloned_original_sequence TTTAGAATATCAGCATCCGTCGCTACCGCCTGTATGCTCCATGGGGCTAGTTCTAGGCCA

************************************************************

NGS_cotton_genome GCCACAGCTAGAAAGAGCTCAGGGTTGAGTGGAACCGTCAGAATCCCTGGGGATAAGAGT

Cloned_original_sequence GCCACAGCTAGAAAGAGCTCAGGGTTGAGTGGAACCGTCAGAATCCCTGGGGATAAGAGT

************************************************************

NGS_cotton_genome ATTTCACACAGATCCTTCATGTTTGGAGGTTTGGCATCCGGAGAAACCCGTATCACCGGG

Cloned_original_sequence ATTTCACACAGATCCTTCATGTTTGGAGGTTTGGCATCCGGAGAAACCCGTATCACCGGG

************************************************************

NGS_cotton_genome CTCCTGGAAGGTGAGGACGTCATTAACACCGGAAAGGCAATGCAGGCTATGGGGGCTCGT

Cloned_original_sequence CTCCTGGAAGGTGAGGACGTCATTAACACCGGAAAGGCAATGCAGGCTATGGGGGCTCGT

************************************************************

NGS_cotton_genome ATTAGAAAAGAAGGTGACACGTGGATTATTGACGGCGTTGGAAATGGAGGTCTTCTCGCT

Cloned_original_sequence ATTAGAAAAGAAGGTGACACGTGGATTATTGACGGCGTTGGAAATGGAGGTCTTCTCGCT

************************************************************

NGS_cotton_genome CCCGAAGCTCCTCTCGACTTCGGTAACGCCGCTACCGGATGCAGATTGACCATGGGTTTG

Cloned_original_sequence CCCGAAGCTCCTCTCGACTTCGGTAACGCCGCTACCGGATGCAGATTGACCATGGGTTTG

************************************************************

NGS_cotton_genome GTTGGTGTTTATGATTTCGACTCAACTTTTATCGGTGATGCATCTTTAACAAAAAGACCT

Cloned_original_sequence GTTGGTGTTTATGATTTCGACTCAACTTTTATCGGTGATGCATCTTTAACAAAAAGACCT

************************************************************

NGS_cotton_genome ATGGGGCGAGTCTTGAATCCATTGAGGGAGATGGGCGTACAAGTTAAATCTGAAGATGGC

Cloned_original_sequence ATGGGGCGAGTCTTGAATCCATTGAGGGAGATGGGCGTACAAGTTAAATCTGAAGATGGC

************************************************************

NGS_cotton_genome GACCGACTGCCAGTCACTCTTAGGGGGCCAAAGACTCCCACTCCCATAACTTATCGTGTT

Cloned_original_sequence GACCGACTGCCAGTCACTCTTAGGGGGCCAAAGACTCCCACTCCCATAACTTATCGTGTT

************************************************************

NGS_cotton_genome CCTATGGCATCAGCACAGGTTAAGTCTGCTGTTCTTTTAGCTGGTCTTAATACACCTGGA

Cloned_original_sequence CCTATGGCATCAGCACAGGTTAAGTCTGCTGTTCTTTTAGCTGGTCTTAATACACCTGGA

************************************************************

NGS_cotton_genome ATCACAACTGTTATTGAGCCCATTATGACATGTGATCATACGGAGAAGATGTTGCAGGGG

Cloned_original_sequence ATCACAACTGTTATTGAGCCCATTATGACATGTGATCATACGGAGAAGATGTTGCAGGGG

************************************************************

NGS_cotton_genome TTCGGAGCCAATTTGACTGTGGAGACAGATGCTGATGGTGTGAGGACTATTAGACTGGAA

Cloned_original_sequence TTCGGAGCCAATTTGACTGTGGAGACAGATGCTGATGGTGTGAGGACTATTAGACTGGAA

************************************************************

NGS_cotton_genome GGACGTGGCAAACTCACCGGTCAAGTTATCGATGTCCCAGGTGATCCTTCCTCTACCGCC

Cloned_original_sequence GGACGTGGCAAACTCACCGGTCAAGTTATCGATGTCCCAGGTGATCCTTCCTCTACCGCC

************************************************************

NGS_cotton_genome TTTCCTTTAGTTGCAGCCCTGCTCGTGCCAGGCTCTGATGTGACCATTCTCAATGTTCTG

Cloned_original_sequence TTTCCTTTAGTTGCAGCCCTGCTCGTGCCAGGCTCTGATGTGACCATTCTCAATGTTCTG

************************************************************

NGS_cotton_genome ATGAACCCAACTAGGACGGGACTTATACTCACACTGCAGGAGATGGGAGCAGATATCGAG

Cloned_original_sequence ATGAACCCAACTAGGACGGGACTTATACTCACACTGCAGGAGATGGGAGCAGATATCGAG

************************************************************

NGS_cotton_genome GTAATAAATCTCCGTCTCGCAGGAGGTGAGGACGTTGCAGATCTTCGTGTTCGTTCCTCT

Cloned_original_sequence GTAATAAATCTCCGTCTCGCAGGAGGTGAGGACGTTGCAGATCTTCGTGTTCGTTCCTCT

************************************************************

NGS_cotton_genome ACATTAAAGGGTGTCACCGTTCCGGAAGATAGGGCCCCACCAATGATTGATGAATACCCT

Cloned_original_sequence ACATTAAAGGGTGTCACCGTTCCGGAAGATAGGGCCCCACCAATGATTGATGAATACCCT

************************************************************

NGS_cotton_genome ATCCTTGCAGTCGCTGCAGCCTTTGCTGAAGGTGCTACAGTAATGAATGGCCTTGAGGAA

Cloned_original_sequence ATCCTTGCAGTCGCTGCAGCCTTTGCTGAAGGTGCTACAGTAATGAATGGCCTTGAGGAA

************************************************************

NGS_cotton_genome CTTAGGGTGAAGGAATCTGATAGGCTCAGTGCAGTGGCTAATGGGTTGAAATTGAATGGT

Cloned_original_sequence CTTAGGGTGAAGGAATCTGATAGGCTCAGTGCAGTGGCTAATGGGTTGAAATTGAATGGT

************************************************************

NGS_cotton_genome GTAGACTGTGACGAGGGTGAAACTTCACTTGTTGTGCGAGGTAGACCCGATGGCAAGGGA

Cloned_original_sequence GTAGACTGTGACGAGGGTGAAACTTCACTTGTTGTGCGAGGTAGACCCGATGGCAAGGGA

************************************************************

NGS_cotton_genome CTTGGCAACGCCTCAGGTGCCGCCGTTGCTACTCACTTGGATCATAGGATTGCTATGAGC

Cloned_original_sequence CTTGGCAACGCCTCAGGTGCCGCCGTTGCTACTCACTTGGATCATAGGATTGCTATGAGC

************************************************************

NGS_cotton_genome TTCTTGGTGATGGGGTTAGTGTCTGAGAACCCGGTGACTGTAGACGATGCAACAATGATC

Cloned_original_sequence TTCTTGGTGATGGGGTTAGTGTCTGAGAACCCGGTGACTGTAGACGATGCAACAATGATC

************************************************************

NGS_cotton_genome GCCACAAGTTTCCCTGAGTTTATGGATCTTATGGCTGGACTCGGTGCCAAAATTGAACTG

Cloned_original_sequence GCCACAAGTTTCCCTGAGTTTATGGATCTTATGGCTGGACTCGGTGCCAAAATTGAACTG

************************************************************

NGS_cotton_genome AGCGATACTAAGGCAGCCTAACCTAGGTTCGAGTATTATGGCATTGGGAAAACTGTTTTT

Cloned_original_sequence AGCGATACTAAGGCAGCCTAACCTAGGTTCGAGTATTATGGCATTGGGAAAACTGTTTTT

************************************************************

NGS_cotton_genome CTTGTACCATTTGTTGTGCTTGTAATTTACTGTGTTTTTTATTCGGTTTTCGCTATCGAA

Cloned_original_sequence CTTGTACCATTTGTTGTGCTTGTAATTTACTGTGTTTTTTATTCGGTTTTCGCTATCGAA

************************************************************

NGS_cotton_genome CTGTGAAATGGAAATGGATGGAGAAGAGTTAATGAATGATATGGTCCTTTTGTTCATTCT

Cloned_original_sequence CTGTGAAATGGAAATGGATGGAGAAGAGTTAATGAATGATATGGTCCTTTTGTTCATTCT

************************************************************

NGS_cotton_genome CAAATTAATATTATTTGTTTTTTCTCTTATTTGTTGTGTGTTGAATTTGAAATTATAAGA

Cloned_original_sequence CAAATTAATATTATTTGTTTTTTCTCTTATTTGTTGTGTGTTGAATTTGAAATTATAAGA

************************************************************

NGS_cotton_genome GATATGCAAACATTTTGTTTTGAGTAAAAATGTGTCAAATCGTGGCCTCTAATGACCGAA

Cloned_original_sequence GATATGCAAACATTTTGTTTTGAGTAAAAATGTGTCAAATCGTGGCCTCTAATGACCGAA

************************************************************

NGS_cotton_genome GTTAATATGAGGAGTAAAACATCGGCGCGCCAAGCTTCATCAACGCAAGACATGCGCACG

Cloned_original_sequence GTTAATATGAGGAGTAAAACATCGGCGCGCCAAGCTTCATCAACGCAAGACATGCGCACG

************************************************************

NGS_cotton_genome ACCGTCTGACAGGAGAGGAATTTCCGACGAGCACAGAAAGGACTTGCTCTTGGACGTAGG

Cloned_original_sequence ACCGTCTGACAGGAGAGGAATTTCCGACGAGCACAGAAAGGACTTGCTCTTGGACGTAGG

************************************************************

NGS_cotton_genome CCTATTTCTCAGGCACATGTATCAAGTGTTCGGACGTGGGTTTTCGATGGTGTATCAGCC

Cloned_original_sequence CCTATTTCTCAGGCACATGTATCAAGTGTTCGGACGTGGGTTTTCGATGGTGTATCAGCC

************************************************************

NGS_cotton_genome GCCGCCAACTGGGAGATGAGGAGGCTTTCTTGGGGGGCAGTCAGCAGTTCATTTCACAAG

Cloned_original_sequence GCCGCCAACTGGGAGATGAGGAGGCTTTCTTGGGGGGCAGTCAGCAGTTCATTTCACAAG

************************************************************

NGS_cotton_genome ACAGAGGAACTTGTAAGGAGATGCACTGATTTATCTTGGCGCAAACCAGCAGGACGAATT

Cloned_original_sequence ACAGAGGAACTTGTAAGGAGATGCACTGATTTATCTTGGCGCAAACCAGCAGGACGAATT

************************************************************

NGS_cotton_genome AGTGGGAATAGCCCGCGAATATCTAAGTTATGCCTGTCGGCATGAGCAGAAACTTCCAAT

Cloned_original_sequence AGTGGGAATAGCCCGCGAATATCTAAGTTATGCCTGTCGGCATGAGCAGAAACTTCCAAT

************************************************************

NGS_cotton_genome TCGAAACAGTTTGGAGAGGTTGTTTTTGGGCATACCTTTTGTTAGTCAGCCTCTCGATTG

Cloned_original_sequence TCGAAACAGTTTGGAGAGGTTGTTTTTGGGCATACCTTTTGTTAGTCAGCCTCTCGATTG

************************************************************

NGS_cotton_genome CTCATCGTCATTACACAGTACCGAAGTTTGATCGATCTAGTAACATAGATGACACCGCGC

Cloned_original_sequence CTCATCGTCATTACACAGTACCGAAGTTTGATCGATCTAGTAACATAGATGACACCGCGC

************************************************************

NGS_cotton_genome GCGATAATTTATCCTAGTTTGCGCGCTATATTTTGTTTTCTATCGCGTATTAAATGTATA

Cloned_original_sequence GCGATAATTTATCCTAGTTTGCGCGCTATATTTTGTTTTCTATCGCGTATTAAATGTATA

************************************************************

NGS_cotton_genome ATTGCGGGACTCTAATCATAAAAACCCATCTCATAAATAACGTCATGCATTACATGTTAA

Cloned_original_sequence ATTGCGGGACTCTAATCATAAAAACCCATCTCATAAATAACGTCATGCATTACATGTTAA

************************************************************

NGS_cotton_genome TTATTACATGCTTAACGTAATTCAACAGAAATTATATGATAATCATCGCAAGACCGGCAA

Cloned_original_sequence TTATTACATGCTTAACGTAATTCAACAGAAATTATATGATAATCATCGCAAGACCGGCAA

************************************************************

NGS_cotton_genome CAGGATTCAATCTTAAGAAACTTTATTGCCAAATGTTTGAACGATCTGCTTCGACGCACT

Cloned_original_sequence CAGGATTCAATCTTAAGAAACTTTATTGCCAAATGTTTGAACGATCTGCTTCGACGCACT

************************************************************

NGS_cotton_genome CCTTCTTTACTCCACCATCTCGTCCTTATTGAAAACGTGGGTAGCACCAAAACGAATCAA

Cloned_original_sequence CCTTCTTTACTCCACCATCTCGTCCTTATTGAAAACGTGGGTAGCACCAAAACGAATCAA

************************************************************

NGS_cotton_genome GTCGCTGGAACTGAAGTTACCAATCACGCTGGATGATTTGCCAGTTGGATTAATCTTGCC

Cloned_original_sequence GTCGCTGGAACTGAAGTTACCAATCACGCTGGATGATTTGCCAGTTGGATTAATCTTGCC

************************************************************

NGS_cotton_genome TTTCCCCGCATGAATAATATTGATGAATGCATGCGTGAGGGGTATTTCGATTTTGGCAAT

Cloned_original_sequence TTTCCCCGCATGAATAATATTGATGAATGCATGCGTGAGGGGTATTTCGATTTTGGCAAT

************************************************************

NGS_cotton_genome AGCTGCAATTGCCGCGACATCCTCCAACGAGCATAATTCTTCAGAAAAATAGCGATGTTC

Cloned_original_sequence AGCTGCAATTGCCGCGACATCCTCCAACGAGCATAATTCTTCAGAAAAATAGCGATGTTC

************************************************************

NGS_cotton_genome CATGTTGTCAGGGCATGCATGATGCACGTTATGAGGTGACGGTGCTAGGCAGTATTCCCT

Cloned_original_sequence CATGTTGTCAGGGCATGCATGATGCACGTTATGAGGTGACGGTGCTAGGCAGTATTCCCT

************************************************************

NGS_cotton_genome CAAAGTTTCATAGTCAGTATCATATTCATCATTGCATTCCTGCAAGAGAGAATTGAGACG

Cloned_original_sequence CAAAGTTTCATAGTCAGTATCATATTCATCATTGCATTCCTGCAAGAGAGAATTGAGACG

************************************************************

NGS_cotton_genome CAATCCACACGCTGCGGCAACCTTCCGGCGTTCGTGGTCTATTTGCTCTTGGACGTTGCA

Cloned_original_sequence CAATCCACACGCTGCGGCAACCTTCCGGCGTTCGTGGTCTATTTGCTCTTGGACGTTGCA

************************************************************

NGS_cotton_genome AACGTAAGTGTTGGATCGGGGTGGGCGAAGAACTCCAGCATGAGATCCCCGCGCTGGAGG

Cloned_original_sequence AACGTAAGTGTTGGATCGGGGTGGGCGAAGAACTCCAGCATGAGATCCCCGCGCTGGAGG

************************************************************

NGS_cotton_genome ATCATCCAGCCGGCGTCCCGGAAAACGATTCCGAAGCCCAACCTTTCATAGAAGGCGGCG

Cloned_original_sequence ATCATCCAGCCGGCGTCCCGGAAAACGATTCCGAAGCCCAACCTTTCATAGAAGGCGGCG

************************************************************

NGS_cotton_genome GTGGAATCGAAATCTCGTGATGGCAGGTTGGGCGTCGCTTGGTCGGTCATTTCGAACCCC

Cloned_original_sequence GTGGAATCGAAATCTCGTGATGGCAGGTTGGGCGTCGCTTGGTCGGTCATTTCGAACCCC

************************************************************

NGS_cotton_genome AGAGTCCCGCTCAGAAGAACTCGTCAAGAAGGCGATAGAAGGCGATGCGCTGCGAATCGG

Cloned_original_sequence AGAGTCCCGCTCAGAAGAACTCGTCAAGAAGGCGATAGAAGGCGATGCGCTGCGAATCGG

************************************************************

NGS_cotton_genome GAGCGGCGATACCGTAAAGCACGAGGAAGCGGTCAGCCCATTCGCCGCCAAGCTCTTCAG

Cloned_original_sequence GAGCGGCGATACCGTAAAGCACGAGGAAGCGGTCAGCCCATTCGCCGCCAAGCTCTTCAG

************************************************************

NGS_cotton_genome CAATATCACGGGTAGCCAACGCTATGTCCTGATAGCGGTCCGCCACACCCAGCCGGCCAC

Cloned_original_sequence CAATATCACGGGTAGCCAACGCTATGTCCTGATAGCGGTCCGCCACACCCAGCCGGCCAC

************************************************************

NGS_cotton_genome AGTCGATGAATCCAGAAAAGCGGCCATTTTCCACCATGATATTCGGCAAGCAGGCATCGC

Cloned_original_sequence AGTCGATGAATCCAGAAAAGCGGCCATTTTCCACCATGATATTCGGCAAGCAGGCATCGC

************************************************************

NGS_cotton_genome CATGGGTCACGACGAGATCCTCGCCGTCGGGCATGCGCGCCTTGAGCCTGGCGAACAGTT

Cloned_original_sequence CATGGGTCACGACGAGATCCTCGCCGTCGGGCATGCGCGCCTTGAGCCTGGCGAACAGTT

************************************************************

NGS_cotton_genome CGGCTGGCGCGAGCCCCTGATGCTCTTCGTCCAGATCATCCTGATCGACAAGACCGGCTT

Cloned_original_sequence CGGCTGGCGCGAGCCCCTGATGCTCTTCGTCCAGATCATCCTGATCGACAAGACCGGCTT

************************************************************

NGS_cotton_genome CCATCCGAGTACGTGCTCGCTCGATGCGATGTTTCGCTTGGTGGTCGAATGGGCAGGTAG

Cloned_original_sequence CCATCCGAGTACGTGCTCGCTCGATGCGATGTTTCGCTTGGTGGTCGAATGGGCAGGTAG

************************************************************

NGS_cotton_genome CCGGATCAAGCGTATGCAGCCGCCGCATTGCATCAGCCATGATGGATACTTTCTCGGCAG

Cloned_original_sequence CCGGATCAAGCGTATGCAGCCGCCGCATTGCATCAGCCATGATGGATACTTTCTCGGCAG

************************************************************

NGS_cotton_genome GAGCAAGGTGAGATGACAGGAGATCCTGCCCCGGCACTTCGCCCAATAGCAGCCAGTCCC

Cloned_original_sequence GAGCAAGGTGAGATGACAGGAGATCCTGCCCCGGCACTTCGCCCAATAGCAGCCAGTCCC

************************************************************

NGS_cotton_genome TTCCCGCTTCAGTGACAACGTCGAGCACAGCTGCGCAAGGAACGCCCGTCGTGGCCAGCC

Cloned_original_sequence TTCCCGCTTCAGTGACAACGTCGAGCACAGCTGCGCAAGGAACGCCCGTCGTGGCCAGCC

************************************************************

NGS_cotton_genome ACGATAGCCGCGCTGCCTCGTCCTGCAGTTCATTCAGGGCACCGGACAGGTCGGTCTTGA

Cloned_original_sequence ACGATAGCCGCGCTGCCTCGTCCTGCAGTTCATTCAGGGCACCGGACAGGTCGGTCTTGA

************************************************************

NGS_cotton_genome CAAAAAGAACCGGGCGCCCCTGCGCTGACAGCCGGAACACGGCGGCATCAGAGCAGCCGA

Cloned_original_sequence CAAAAAGAACCGGGCGCCCCTGCGCTGACAGCCGGAACACGGCGGCATCAGAGCAGCCGA

************************************************************

NGS_cotton_genome TTGTCTGTTGTGCCCAGTCATAGCCGAATAGCCTCTCCACCCAAGCGGCCGGAGAACCTG

Cloned_original_sequence TTGTCTGTTGTGCCCAGTCATAGCCGAATAGCCTCTCCACCCAAGCGGCCGGAGAACCTG

************************************************************

NGS_cotton_genome CCCGGATCCGGGCGGAAATAGGTAAAGAAGTTGCGGATAAGGTAATTGCCATTGCAGATT

Cloned_original_sequence CCCGGATCCGGGCGGAAATAGGTAAAGAAGTTGCGGATAAGGTAATTGCCATTGCAGATT

************************************************************

NGS_cotton_genome ATTTGGATTGAGAGTGAATATGAGACTCTAATTGGATACCGAGGGGAATTTATGGAACGT

Cloned_original_sequence ATTTGGATTGAGAGTGAATATGAGACTCTAATTGGATACCGAGGGGAATTTATGGAACGT

************************************************************

NGS_cotton_genome CAGTGGAGCATTTTTGACAAGAAATATTTGCTAGCTGATAGTGACCTTAGGCGACTTTTG

Cloned_original_sequence CAGTGGAGCATTTTTGACAAGAAATATTTGCTAGCTGATAGTGACCTTAGGCGACTTTTG

************************************************************

NGS_cotton_genome AACGCGCAATAATGGTTTCTGACGTATGTGCTTAGCTCATTAAACTCCAGAAACCCGCGG

Cloned_original_sequence AACGCGCAATAATGGTTTCTGACGTATGTGCTTAGCTCATTAAACTCCAGAAACCCGCGG

************************************************************

NGS_cotton_genome CTGAGTGGCTCCTTCAACGTTGCGGTTCTGTCAGTTCCAAACGTAAAACGGCTTGTCCCG

Cloned_original_sequence CTGAGTGGCTCCTTCAACGTTGCGGTTCTGTCAGTTCCAAACGTAAAACGGCTTGTCCCG

************************************************************

NGS_cotton_genome CGTCATCGGCGGGGGTCATAACGTGACTCCCTTAATTCTCCGCTCATGATCAGATTGTCG

Cloned_original_sequence CGTCATCGGCGGGGGTCATAACGTGACTCCCTTAATTCTCCGCTCATGATCAGATTGTCG

************************************************************

**^Cotton genome sequence^**

NGS_cotton_genome TTTCCCGCCTTCAGTTT

Cloned_original_sequence TTTCCCGCCTTCAGTTT

*****************
